# Network-neuron interactions underlying sensory responses of layer 5 pyramidal tract neurons in barrel cortex

**DOI:** 10.1371/journal.pcbi.1011468

**Published:** 2024-04-16

**Authors:** Arco Bast, Rieke Fruengel, Christiaan P. J. de Kock, Marcel Oberlaender

**Affiliations:** 1 *In Silico* Brain Sciences Group, Max Planck Institute for Neurobiology of Behavior ˗ caesar, Bonn, Germany; 2 International Max Planck Research School (IMPRS) for Brain and Behavior, Bonn, Germany; 3 Department of Integrative Neurophysiology, Center for Neurogenomics and Cognitive Research, Vrije Universiteit Amsterdam, Amsterdam, the Netherlands; Ernst-Strungmann-Institut, GERMANY

## Abstract

Neurons in the cerebral cortex receive thousands of synaptic inputs per second from thousands of presynaptic neurons. How the dendritic location of inputs, their timing, strength, and presynaptic origin, in conjunction with complex dendritic physiology, impact the transformation of synaptic input into action potential (AP) output remains generally unknown for *in vivo* conditions. Here, we introduce a computational approach to reveal which properties of the input causally underlie AP output, and how this neuronal input-output computation is influenced by the morphology and biophysical properties of the dendrites. We demonstrate that this approach allows dissecting of how different input populations drive *in vivo* observed APs. For this purpose, we focus on fast and broadly tuned responses that pyramidal tract neurons in layer 5 (L5PTs) of the rat barrel cortex elicit upon passive single whisker deflections. By reducing a multi-scale model that we reported previously, we show that three features are sufficient to predict with high accuracy the sensory responses and receptive fields of L5PTs under these specific *in vivo* conditions: the count of active excitatory versus inhibitory synapses preceding the response, their spatial distribution on the dendrites, and the AP history. Based on these three features, we derive an analytically tractable description of the input-output computation of L5PTs, which enabled us to dissect how synaptic input from thalamus and different cell types in barrel cortex contribute to these responses. We show that the input-output computation is preserved across L5PTs despite morphological and biophysical diversity of their dendrites. We found that trial-to-trial variability in L5PT responses, and cell-to-cell variability in their receptive fields, are sufficiently explained by variability in synaptic input from the network, whereas variability in biophysical and morphological properties have minor contributions. Our approach to derive analytically tractable models of input-output computations in L5PTs provides a roadmap to dissect network-neuron interactions underlying L5PT responses across different *in vivo* conditions and for other cell types.

## Author summary

Revealing how synaptic inputs drive action potential output is one of the major challenges in neuroscience research. An increasing number of approaches therefore seek to combine detailed measurements at synaptic, cellular and network scales into biologically realistic brain models. Indeed, such models have started to make empirically testable predictions about the inputs that underlie *in vivo* observed activity patterns. However, the enormous complexity of these models generally prevents the derivation of interpretable descriptions that explain how neurons transform synaptic input into action potential output, and how these input-output computations depend on synaptic, cellular and network properties. Here we introduce an approach to reveal input-output computations that neurons in the cerebral cortex perform upon sensory stimulation. For this purpose, we reduce a realistic multi-scale model of the rat barrel cortex to the minimal description that accounts for *in vivo* observed responses to whisker stimuli. Thereby, we identify the input-output computation that these cortical neurons perform under this *in vivo* condition, and we show that this computation is preserved across neurons despite morphological and biophysical diversity. Our approach provides analytically tractable and hence interpretable descriptions of neuronal input-output computations during specific *in vivo* conditions.

## Introduction

Dissecting how neurons transform synaptic input into action potential (AP) output is a prerequisite for understanding the neurobiological implementation of brain functions. Many studies have investigated the principles of synaptic integration for dozens of dendritic stimulus sites [1–4]. However, in the cerebral cortex, pyramidal neurons receive synaptic input from thousands of neurons along their morphologically extensive and biophysically complex dendrites, which they then transform into APs. When and where synapses are active on the dendrite, the ‘spatiotemporal input patterns’, is highly variable from cell to cell, and even from trial to trial within the same experimental condition [5–9]. As a result, which features of these spatiotemporal synaptic input patterns determine AP output *in vivo*, how this transformation depends on variability in morphological and biophysical properties, and which mathematical operation the neuron performs–i.e., what is the ‘input-output computation’–remains unclear.

Here, we derive the input-output computation that layer 5 pyramidal tract neurons (L5PTs) in the vibrissae-related part of the rat primary somatosensory cortex (vS1)–the barrel cortex [10]–perform *in vivo* to transform synaptic inputs evoked upon passive single whisker deflections into AP output. Along their extensive and biophysically complex dendrites, these major cortical output cells receive synaptic input patterns from different thalamocortical, intracortical and top-down corticocortical pathways (reviewed in [11]). In response to sensory stimulation, L5PTs generate fast and reliable AP output with receptive fields that show large cell-to-cell variability and which are broader than those of any other cell type in the same cortical column [12].

How do these AP responses arise from the interplay between neuron and network properties? How does the transformation of synaptic input into AP output depend on specific morphological and biophysical properties of the dendrite? Joint measurement of sensory-evoked synaptic input patterns (i.e., precise time points, dendritic locations, and origins of all active synapses), the morphology of the dendrite, its biophysical properties (which ion channels are active when and where on the dendrite) and AP output would be ideal to answer these questions, but are difficult to assess experimentally. Therefore, we had previously reported and validated a multi-scale model of the rat barrel cortex, which provides realistic estimates for the number and locations of synaptic inputs that impinge onto L5PT dendrites upon whisker stimulation, and how L5PTs transform these inputs into AP output [13–15]. Simulations of the multi-scale model captured the fast and broadly tuned sensory responses of L5PTs *in vivo*, provided predictions about the cellular and circuit mechanisms underlying these responses, and thereby provided concrete predictions for how to test these mechanisms experimentally, which we did successfully via pharmacological manipulations *in vivo* [14]. Thus, the multi-scale model sets the stage to investigate which input-output computation L5PTs perform upon sensory stimulation, and how this computation depends on the morphological and biophysical properties of the dendrites.

Here we address these questions by introducing an approach that seeks to reduce the multi-scale models into analytically tractable models that capture the input-output computations of L5PTs upon single whisker deflections, while maintaining the *in vivo* observed trial-to-trial and cell-to-cell variability. The reduction revealed that the input-output computation of L5PTs can be explained by three features: the count of active synapses in a time window, their soma-distance-dependent spatial distribution on the dendrite, and the time since the previous AP of the L5PT. While the multi-scale model is expressed in partial differential equations, which require numerical solvers to evaluate, the reduced models express the transformation of synaptic input into AP output in an analytically tractable manner. This increased interpretability hence allows dissecting the network-neuron interactions underlying sensory responses of L5PTs for the investigated *in vivo* condition.

## Results

**Fig 1:**
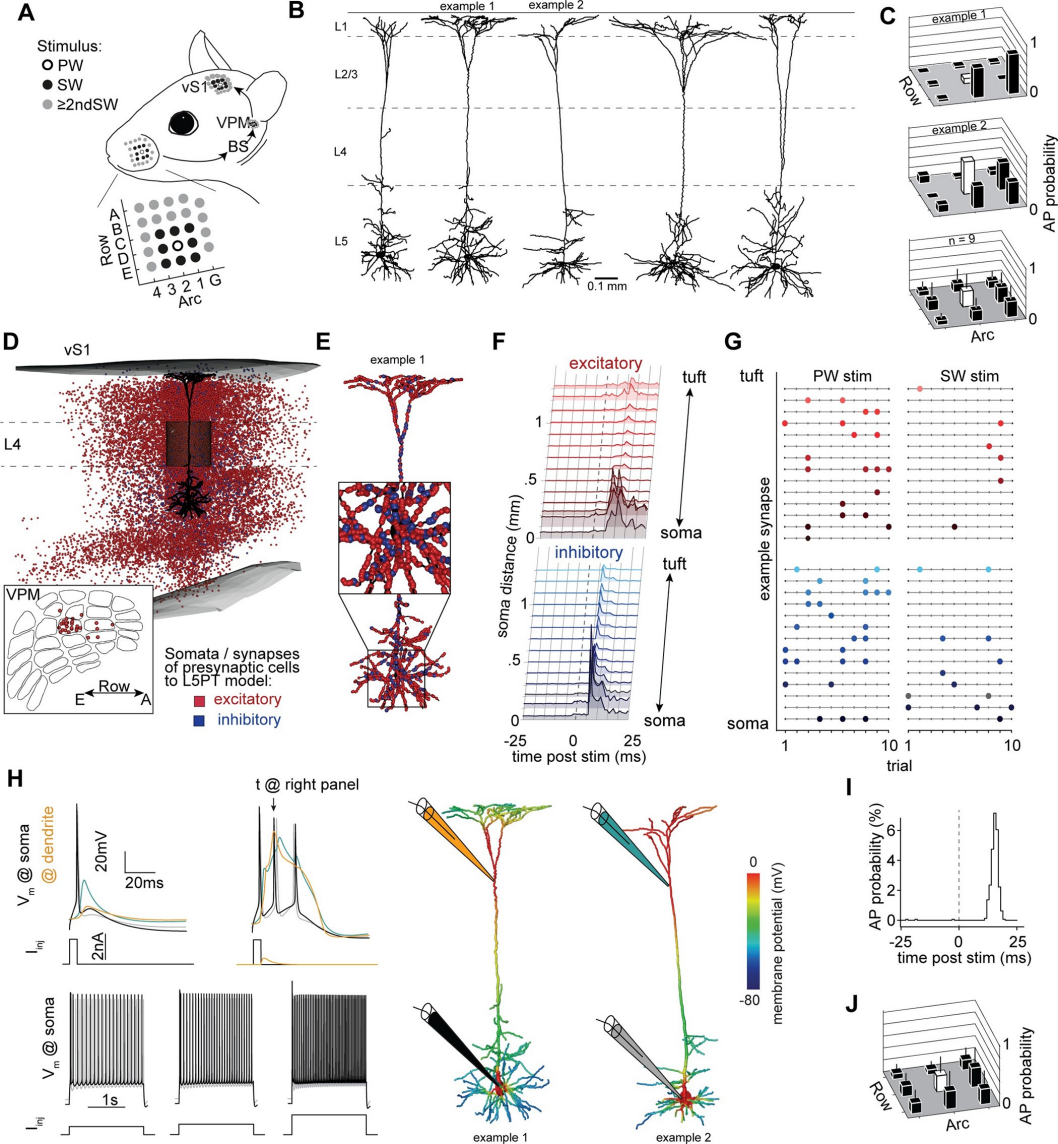
Biophysically detailed multi-scale model of whisker deflection evoked responses in cortical pyramidal tract neurons. **A:** Sensory-evoked signal flow: stimuli of single whiskers (which are arranged in ‘arcs’ and ‘rows’ on the animal’s snout) are transmitted to the brainstem (BS), from there to the VPM thalamus, and from there to the primary sensory cortex of the vibrissal system (vS1). This pathway is somatotopically organized, with barreloids in VPM and barrels in vS1 corresponding to the respective whiskers. **B:** *In vivo* labeled L5PT dendrite morphologies used in this study. **C:** Corresponding receptive fields to passive single whisker touch, measured *in vivo* (upper panels). Average receptive field across 9 *in vivo* recorded L5PTs (lower panel). Error bars are std. **D:** Network model of rat vS1 and VPM provides anatomically realistic estimates of which neurons are connected to a L5PT embedded into the network. In this study, the simulated neurons are located in the C2 column of vS1, thus we refer to the somatotopically aligned C2 whisker as the ‘principal’ whisker, and the adjacent whiskers as surround whiskers. Red and blue markers denote soma locations of presynaptic excitatory and inhibitory somata, respectively. **E:** Synapse distribution originating from the neuron shown in Panel B. **F:** Spatiotemporal input pattern to L5PT: combining the anatomical constraints with empirical measurements of the activity of different presynaptic populations (S1 Fig) provides spatiotemporal input patterns that the L5PT can receive after sensory stimulation. **G:** Trial-to-trial activity of example synapses matching the soma distance from panel F for a principal whisker (C2) and surround whisker (D2) stimulus. **H:** Biophysically detailed multi-compartmental L5PT models reproduce the cell type’s characteristic electrophysiology (left panel), i.e. back propagation of APs (upper left), dendritic Ca-APs and somatic burst firing (upper right), as well as regular firing properties (lower row). Right panel: biophysically detailed neuron morphologies at the moment of a dendritic Ca AP. **I:** Simulated response to principal whisker touch. **J:** Simulated receptive fields across morphologically and biophysically diverse L5PT multi-compartmental models across 81 network embeddings capture broad and heterogeneous receptive fields.

We extended our previously reported multi-scale modelling approach [14] to account for cell-to-cell and trial-to-trial variability. For this purpose, we repeated our simulations of L5PT responses to passive deflections of nine different whiskers, the somatotopically aligned, so-called principal whisker (PW), and any of the eight surrounding whiskers (SW) (**Fig 1A**). Whereas our previous simulations were performed on the morphology of a single *in vivo* recorded L5PT, we now performed simulations for five morphologically diverse L5PTs (**Fig 1B**) for which we had measured highly variable receptive fields *in vivo* (**Fig 1C**). We embedded these morphologies into the network model of the barrel cortex to provide realistic estimates for which neurons in thalamus and barrel cortex (**Fig 1D**) could provide input to these *in vivo* recorded L5PTs, and where along their dendrites these inputs could occur (**Fig 1E**). The network model thereby provided the spatial distributions of synaptic input patterns to L5PTs from different types of excitatory and inhibitory neurons across all layers of the barrel cortex, and from the ventral posterior medial nucleus (VPM)–the primary thalamus of the whisker system. We embedded each morphology at eighty-one locations (**S1 Fig**) in and around the barrel column of the network model that represents the C2 whisker, and activated presynaptic neurons in the network model according to cell type- and layer-specific experimental recording data from passive single whisker deflections (**S2 Fig**). Thereby, for each morphology, each location of the network embedding, and each configuration of active neurons in the network, we generate a unique but empirically well-constrained spatiotemporal synaptic input pattern to L5PT dendrites (**Fig 1F**). The hence predicted spatiotemporal synaptic input patterns (**Fig 1G**) are remarkably consistent with those observed empirically via dendritic spine imaging [9].

To simulate how such spatiotemporal input patterns are transformed into AP output, we converted the L5PTs into biophysically detailed multi-compartmental models (n = 7 from 5 morphologies), which capture their characteristic electrophysiological properties [16], including backpropagating APs, dendritic calcium APs, and responses to step current injections (**Fig 1H and S1 Table**). Each multi-compartmental model used different biophysical parameters to achieve these electrophysiological properties, reflecting different densities of active conductances in different subcellular compartments (**S2 Table**). Thus, the embedding of these diverse multi-compartmental models into the network model allowed us to investigate how variability in synaptic input, in conjunction with variability in morphological and biophysical properties of the dendrites, impact sensory responses of L5PTs. Simulations of each of these multi-scale model configurations predicted the characteristic fast responses (**Fig 1I**) and broad receptive fields of L5PTs (**Fig 1J**). Moreover, the variability of *in silico* responses across multi-scale model configurations matched the cell-to-cell variability observed *in vivo* across L5PTs: The distribution of response probabilities closely matches for any whisker (p-values ranging between 0.07 and 0.92) and the means of these distributions, i.e., the ‘mean receptive field’ is significantly correlated (R value 0.76, p = 0.017). Thus, these multi-scale model configurations set the stage to investigate which features of synaptic input patterns determine AP output, and how this transformation depends on variability in morphological and biophysical properties of the dendrites.

### Input-output computation underlying sensory responses of L5PTs

**Fig 2:**
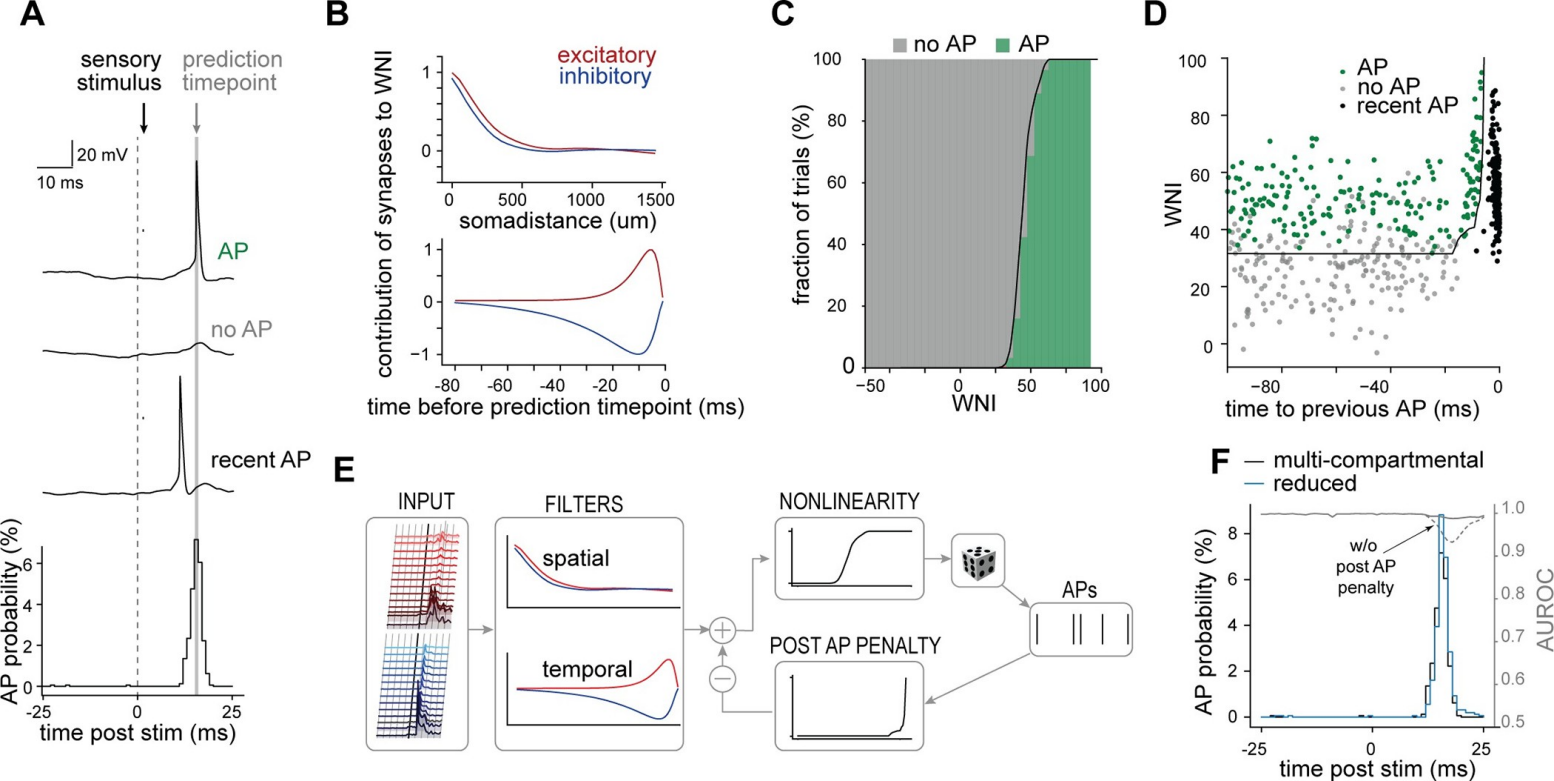
Input-output computation of L5PTs upon single whisker deflections. **A:** Exemplary responses of the multi-compartmental model with respect to the prediction time point (the time point for which the occurrence of an AP is to be predicted) for the three relevant response categories ‘AP’, ‘no AP’, and ‘recent AP’ (AP was elicited shortly before the prediction time point). **B:** Spatiotemporal input filter that best separates AP and no AP trials assigns strong weight to proximal synapses (top) active in a short time window before the prediction time point (bottom). **C:** Nonlinear relationship between WNI and AP probability. WNI represents the ‘drive’ a neuron receives; the higher the WNI the higher the probability an AP will be generated. **D:** Weighted net input–the input filtered by the spatiotemporal filter–separates AP and no AP trials, but not ‘recent AP’ trials, which can be distinguished based on a second measure, ‘time to previous AP’.**E:** Reduced model structure. APs are generated stochastically based on the AP probability (output of the nonlinearity). If an AP is generated, subsequent APs become less likely due to the post AP penalty, which is subtracted from the WNI. This reduced model directly relates AP output to synaptic input and previously generated APs in the simulated *in vivo* condition. **F:** The reduced model’s responses match the biophysically detailed model across many trials (close PSTH match) and on the single trial level (high AUROC score across all time points). Without the post AP penalty, the AUROC score drops during the sensory-evoked response.

We developed our reduction approach by using one of the multi-compartmental models, whose morphology is shown as example 1 in Fig 1B. For each of its eighty-one network embeddings, we simulated its responses to passive deflections of the PW and the eight SWs, respectively. Within this simulation data, we searched for features in the synaptic input that are most predictive for the generation of an AP at a given millisecond—the ‘prediction time point’ (**Fig 2A**, methods). For this purpose, we grouped synapses according to their activation time points (1ms bins), by their pathlength distance to the soma (50μm bins), and depending on whether they are excitatory or inhibitory. We found that a weighted count of active excitatory versus inhibitory synapses, where the contribution of each synapse is weighted depending on its soma distance and activation time point, can predict AP output. We therefore determined the spatial and temporal weights (the “spatiotemporal filter”) that maximize the AP output prediction accuracy (methods). According to these weights (**Fig 2B**), the contributions of both excitatory and inhibitory inputs to AP output decay gradually with soma distance, reaching approximately zero at 500μm distance. Hence, primarily inputs to the proximal (i.e., basal and apical oblique) dendrites of L5PTs are predicted to contribute to their AP output upon sensory stimulation under this specific *in vivo* condition. In the temporal dimension, the contribution of excitatory and inhibitory synapses has a time course resembling excitatory and inhibitory postsynaptic potentials (EPSPs and IPSPs). Excitatory and inhibitory synapses contribute the most to AP output if they were active around 4ms and 9ms before the prediction time point, respectively. These observations were robust, independent of the method used for estimating the weights (methods, **S3 Fig**). The probability to observe a sensory-evoked AP in the multi-scale simulations increased with this weighted count of active synapses (**Fig 2C**), which we in the following refer to as ‘weighted net input’ (WNI). The WNI predicted AP output with high accuracy, as we quantified by the area under the receiver operating characteristic curve (AUROC 0.949).

We revisited trials which were misclassified by the WNI and found that misclassifications mostly occur if there was a recent AP a few milliseconds before the prediction time point. We found that the multi-compartmental model is less excitable shortly after an AP, reflecting time constants of the involved ion channels, and the chance of eliciting an AP is hence low, even if the WNI is high. We therefore distinguished three categories: no AP, AP and recent AP (**Fig 2A**). We show that these three categories can be separated based on the WNI and time to previous AP (**Fig 2D**). To include this AP history-dependent relationship, we determined the separating line of response trials from the others (**Fig 2D** black line, methods) and incorporated it as a ‘post AP penalty’, i.e., we first computed the WNI as before and then subtracted a penalty value depending on the time since the previous AP. This penalized WNI predicted AP output with higher accuracy (AUROC 0.990) than WNI alone. Thus, three features, the spatial and temporal component of the WNI and the post AP penalty are sufficient to predict AP output from synaptic input in the multi-scale models.

Based on these three features, we could hence describe the input-output computation of the biophysically-detailed multi-compartmental model by an analytically tractable model. For this purpose, we assembled post AP penalty and the nonlinear relationship between WNI and AP probability into a generalized linear model (GLM, **Fig 2E**). We applied the GLM to predict AP output at different time points ranging from 25ms before to 25ms after the onset of sensory stimulation. Notably, even though the GLM was developed to predict the peak of AP responses, it also maintained a high AUROC score before and after this time point (**Fig 2F**). On a single-trial level, the model was able to predict if an AP was elicited in the 25ms after the sensory stimulus with an accuracy of 96.7% (**S3 Table**), and the timing of APs within this window was accurate to within 1.1±1.6ms (**S4 Fig**). Thus, the GLM accurately predicts APs throughout the entire 50ms time interval, and the post-stimulus time histogram (PSTH) predicted by the GLM hence matched with the PSTH predicted by the multi-compartmental model (**Fig 2F**).

We investigated how the performance of the GLM depends on the three features it is based on. Without incorporating the AP history (i.e., post AP penalty) into the GLM, the AUROC score drops during the peak response (**Fig 2F**, dashed line). Furthermore, when we simplified the GLM to neglect the spatial dimension, such that the weighted count only considers the time point of activation, but not the soma distance, the AUROC score decreased (**S3A and S3E Fig**). It was also insufficient to incorporate the spatial dimension categorically by only distinguishing a proximal and a distal compartment (**S3B and S3E Fig**). Therefore, soma distance and synapse activation time point need to be included at high resolution (50μm spatial bins, 1ms temporal bins) to accurately capture the input-output computation. To investigate the relative importance of the temporal and spatial filters on the prediction accuracy of the reduced model, we replaced one of these filters at a time with a fixed filter that only provides a cutoff value but does not weight synapses: replacing the spatial filter with weights of 1 for all inputs with less than 500μm pathlength distance from the soma and 0 otherwise reduced the AUROC score to 0.955. Similarly, modifying the temporal filter by assigning weights of 1 and -1 to excitatory and inhibitory synapses that were active in the 50ms preceding the AP, respectively, and setting the weights to 0 for all other active synapses reduced the AUROC score to 0.959. The modifications indicate that the spatial and temporal components of the WNI contribute equally to the prediction accuracy of the reduced model. In turn, considering the specific cell types of the neurons from which the synaptic inputs originate did not increase the prediction accuracy (**S3C–S3E Fig**). Taken together, the AP history, soma-distance dependent spatial distribution of synapses and their temporal activation pattern are necessary and sufficient features to accurately predict APs in the investigated L5PT multi-scale model. Thus, reduced models that are based on these three features, such as the GLM described here, provide an analytically tractable description of the input-output computation that a L5PT with this morphology and these biophysical properties performs upon single whisker deflections.

### Input-output computation is robust to morphological and biophysical diversity

**Fig 3:**
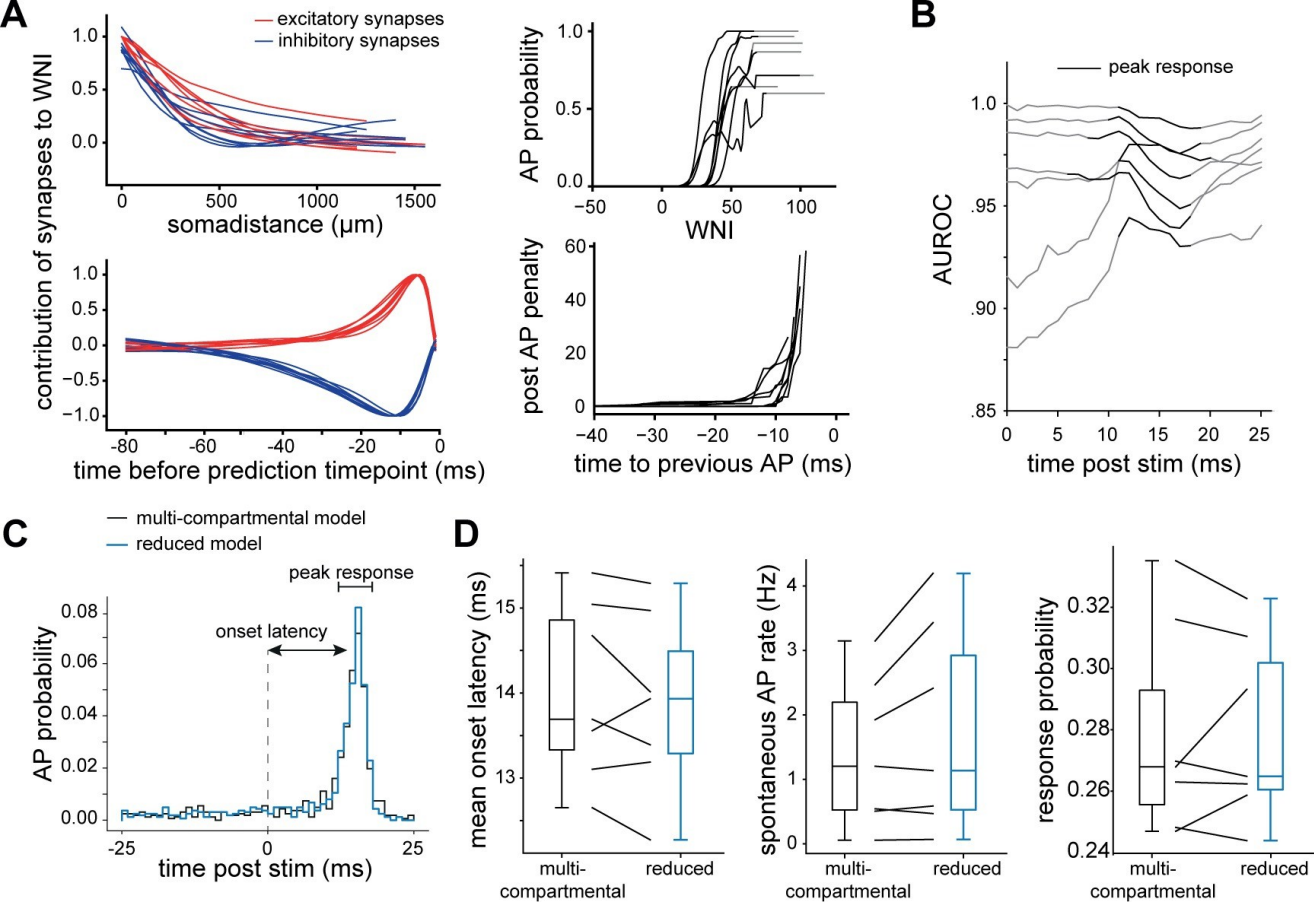
Input-output computation is robust to morphological and biophysical diversity. **A:** Reduced models inferred on the different multi-compartmental models are qualitatively similar, with similar temporal and spatial filters, nonlinearity and post AP penalty. **B:** All models have high AUROC scores, specifically during the sensory-evoked (peak) response. **C:** We quantify latency, spontaneous AP rate (before the stimulus) and response probability for each pair of multi-compartmental and reduced model. **D:** Comparing response properties between multi-compartmental and corresponding reduced model shows close match.

How does this input-output computation depend on the morphological and biophysical properties of L5PTs? To address this question, we applied the reduction strategy to all multi-scale model configurations of L5PTs with morphologically and biophysically diverse dendrites. The reduction revealed that the shape of the spatiotemporal filters, the shape of the penalty and nonlinearity are qualitatively similar across all L5PTs despite their morphological and biophysical diversity (**Fig 3A**): all L5PTs count active synapses, strongly weighting proximal input that occurs within the characteristic EPSP/IPSP-like time windows, with a similar AP-history dependent penalty. Each reduced model achieved a high accuracy before and after the sensory stimulus (**Fig 3B**), and highest accuracies during the peak response (AUROC median/min/max: 0.97/0.93/0.998). At single trial level, the models were able to predict if an AP was elicited in the 25ms after the sensory stimulus with a mean accuracy of 90.9% ± 3.9% (min 85.0%, max 96.7%, confusion matrices for all models are provided in **S3 Table**) and the timing of AP times was predicted accurately to within a mean of 2.0ms (min 1.1ms, max 3.1ms, **S4 Fig**). Thus, this input-output computation is robust across morphologically and biophysically diverse L5PTs.

Notably, while the properties of the reduced models are qualitatively similar, they are not identical. For example, the decay of the spatial filter with soma distance differs slightly between reduced models, which largely reflects diameter differences of the apical trunk dendrite (**S5 Fig**). To what degree do these small variations in the reduced model capture differences of the input-output computations across L5PTs? We compared the predicted PSTHs between the multi-scale configurations of each multi-compartmental model with the respective reduced model and found that the slight differences between their shapes are captured well by the reduced model (**S4 Fig**). To quantify this similarity, we compared the time to maximum response (latency), AP rate before the stimulus, and response probability between multi-compartmental models and corresponding reduced models (**Fig 3C**). Each reduced model generated responses which matched those of the corresponding multi-compartmental models in all of these properties, while preserving the considerable variation across them (**Fig 3D**). Thus, the small variabilities in the reduced models account for differences in the input-output computation between the multi-compartmental models.

The model structure that we found to accurately predict AP output in simulations of the multi-compartmental models is surprisingly linear. This indicates that synaptic input patterns that mimic passive single whisker deflections during our specific *in vivo* condition do either not strongly activate nonlinear dendritic mechanisms in L5PTs, or these mechanisms are activated, but do not strongly influence the prediction of AP output. To test these possibilities, we quantified the occurrences of calcium APs and sodium currents in the dendrites, and the contributions of NMDA and AMPA currents in apical and basal dendrites, respectively. For this purpose, we recorded currents through voltage-gated ion channels, and through AMPA and NMDA receptors during simulations of PW deflections in a multi-compartmental model (example morphology 2). We recorded these currents for 16 locations across the basal and apical dendrites, including the primary branchpoint of the apical trunk (**S6A Fig**). Dendritic nonlinear mechanisms were frequently activated in the simulations. For example, in the apical tuft, we found peaks of sodium influx (**S6E Fig**). At the primary branchpoint, we found that calcium APs occurred in 18% of the simulation trials (**S6B Fig**). The calcium APs, however, did generally not result in somatic burst firing (response during 179 trials with calcium APs: 13% no AP, 83% 1 AP, 4% burst with 2 APs). The reduced model predicted the response equally well for trials with and without calcium APs (86% vs 90%, p = 0.07). Moreover, in our simulations, NMDA currents dominate in apical trunk and tuft dendrites (**S6C, S6D and S6F Fig**). We compared the prediction accuracy between trials with low NMDA contribution (33^rd^ percentile and below) and high NMDA contribution (66^th^ percentile and above) as measured in the tuft dendrites. The strength of these distal NMDA contributions did not affect the prediction accuracy of the reduced models (88% vs 91% for trials with low vs high NMDA contributions, p = 0.17). Thus, nonlinear synaptic and dendritic mechanisms are frequently activated in the simulations that mimic synaptic inputs to L5PTs upon passive single whisker deflections, but either their impact on AP output is low in the investigated condition, or the reduced models are able to capture their impact on the effective input-output computation. To investigate this, we re-simulated all whisker-evoked responses for one multi-compartmental model (example morphology 2) and reduced by 50% or removed NMDA conductance in all synapses. Reducing NMDA reduced response probabilities to all whiskers (**S7A and S7B Fig**), indicating that NMDA strongly contributes to AP output. When we inferred reduced models (**S7C Fig**) from these modified multi-compartmental models, we found that reducing NMDA changes both the spatial and temporal filters. More specifically, NMDA is predicted to increase the influence of distal synapses **(S7D Fig)** and to increase the integration time window **(S7E Fig)**. Notably, nonlinear effects of NMDA thereby decrease the prediction accuracy of the linear reduced models (**S7F Fig**), which hence reach a maximal AUROC score of 0.99999 in the absence of NMDA.

### Network vs neuron contributions to receptive field variability

**Fig 4:**
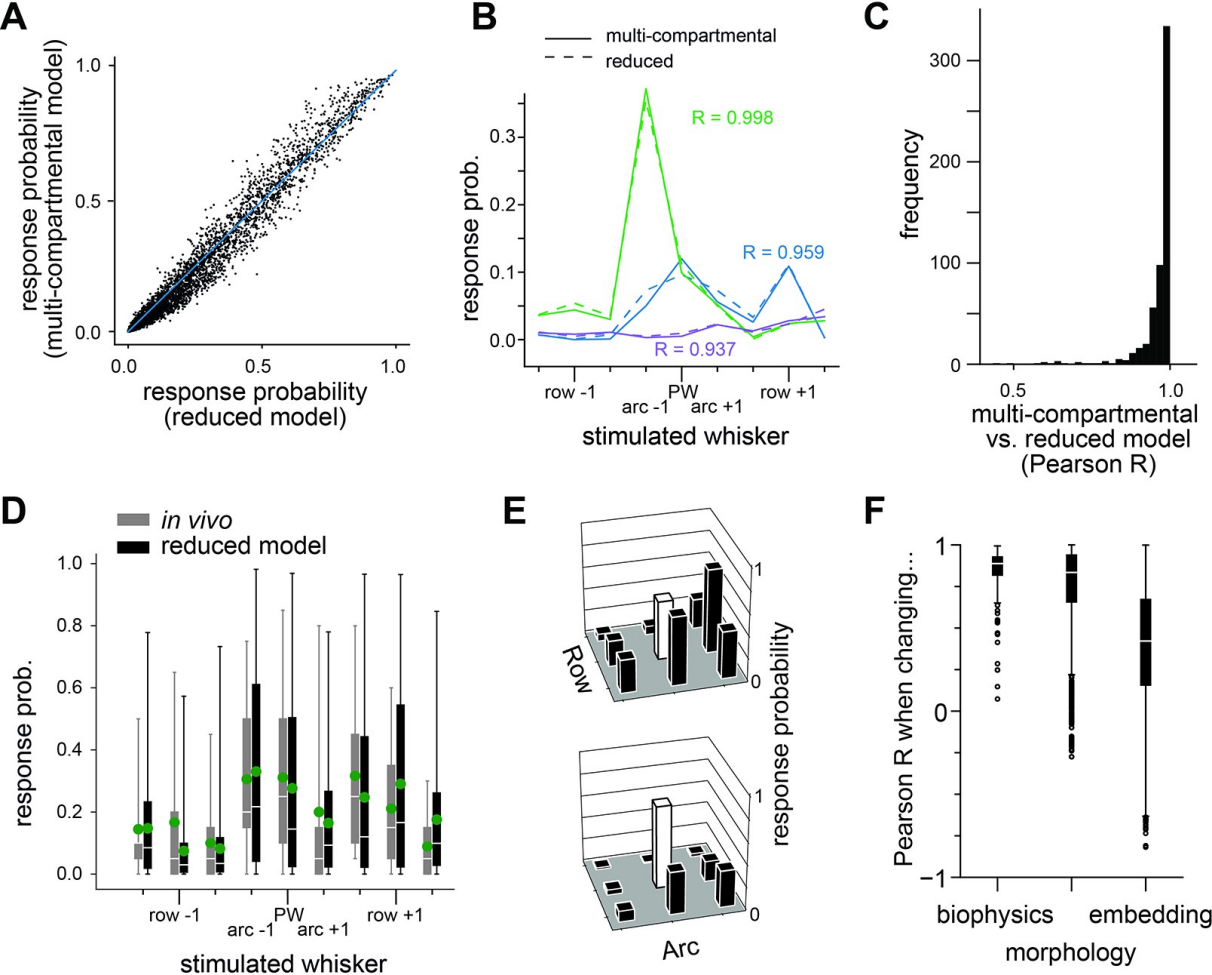
Reduced models predict origins of receptive field variability. **A:** Comparison of responses of 7 different multi-compartmental models and their corresponding reduced models to 9 different whisker stimuli (PW and 8 SW) in 81 different network embedding locations. Response probability is the probability that one or more APs are generated 0-25ms after the sensory stimulus. **B:** Comparison of exemplary receptive field shapes shows close match between biophysically detailed and reduced model. **C:** Quantification of receptive field similarity for all cell positions and biophysically detailed models. **D:** Comparison between *in vivo* and reduced model responses to 9 different whisker stimuli (PW and 8 SW). Green dots represent the mean response probability. **E:** Exemplary receptive fields of reduced models. **F:** Influence of biophysics, morphology and cell position on receptive field shape, quantified by computing the correlation coefficient between receptive fields if one of these properties is changed.

How can the large variability in L5PT responses arise from such small variability in input-output computations? We repeated the simulations of all multi-scale model configurations, this time with the reduced models instead of the multi-compartmental models. The probabilities that a network input elicited a response (i.e., ≥ 1 AP 0-25ms post passive whisker deflection) in the reduced and corresponding multi-compartmental models were highly correlated for input from each whisker, all network embeddings, and all L5PT models (**Fig 4A**, Pearson correlation coefficient = 0.97). Consequently, also the receptive fields were virtually identical between the reduced and multi-compartmental models (Pearson R between receptive fields 0.96±0.01, **Fig 4B and 4C**). Thus, the reduced models capture the variability of responses for each multi-compartmental model across simulation trials, and the variability of responses across multi-compartmental models with different network embeddings, biophysical properties, and/or dendrite morphologies.

How well do the sensory responses predicted by the reduced models match with the *in vivo* data? We compared response probabilities to each whisker stimulus (PW and 8 SWs) between *in vivo* data and *in silico* predictions (**Fig 4D**). The distribution of response probabilities closely matches for any whisker (p values ranging between 0.08 and 0.77) and the means of these distributions, i.e., the ‘mean receptive field’, is significantly correlated (R value 0.77, p = 0.015). The simulated receptive fields even contain matches to the extreme cases observed *in vivo*, for example weaker response to the PW than to a SW, selective response to few whiskers, unselective response to virtually all whiskers (**Fig 4E**). Thus, the reduced models predict means, variance and outliers consistent with those observed *in vivo*, despite the small variability in input-output computations.

Because they capture trial-to-trial and cell-to-cell variability of sensory responses as observed across L5PTs *in vivo*, the reduced models can dissect how variations in network input or neuron properties could contribute to these observations. For this purpose, we computed the pairwise correlation between receptive fields across different morphologies, biophysical parameters, and network embeddings (**Fig 4F**). We found a change in network embedding, and therefore synaptic input patterns, to have the strongest effect (i.e., leads to the largest drop in the Pearson correlation coefficient), followed by the neuron’s morphology and biophysical properties. The models thereby indicate that variations in network input are the primary determinant of receptive field variability from cell to cell, whereas variations in morphology and biophysics play a minor role. Thus, the reduced models predict that L5PTs with diverse morphologies and biophysical properties perform the same input-output computation upon passive single whisker deflections in anesthetized rats, and the variability of their responses across trials and cells is determined by the variability in input that they receive from the network–i.e., from VPM thalamus and barrel cortex.

### Contribution of different input pathways to sensory responses

**Fig 5:**
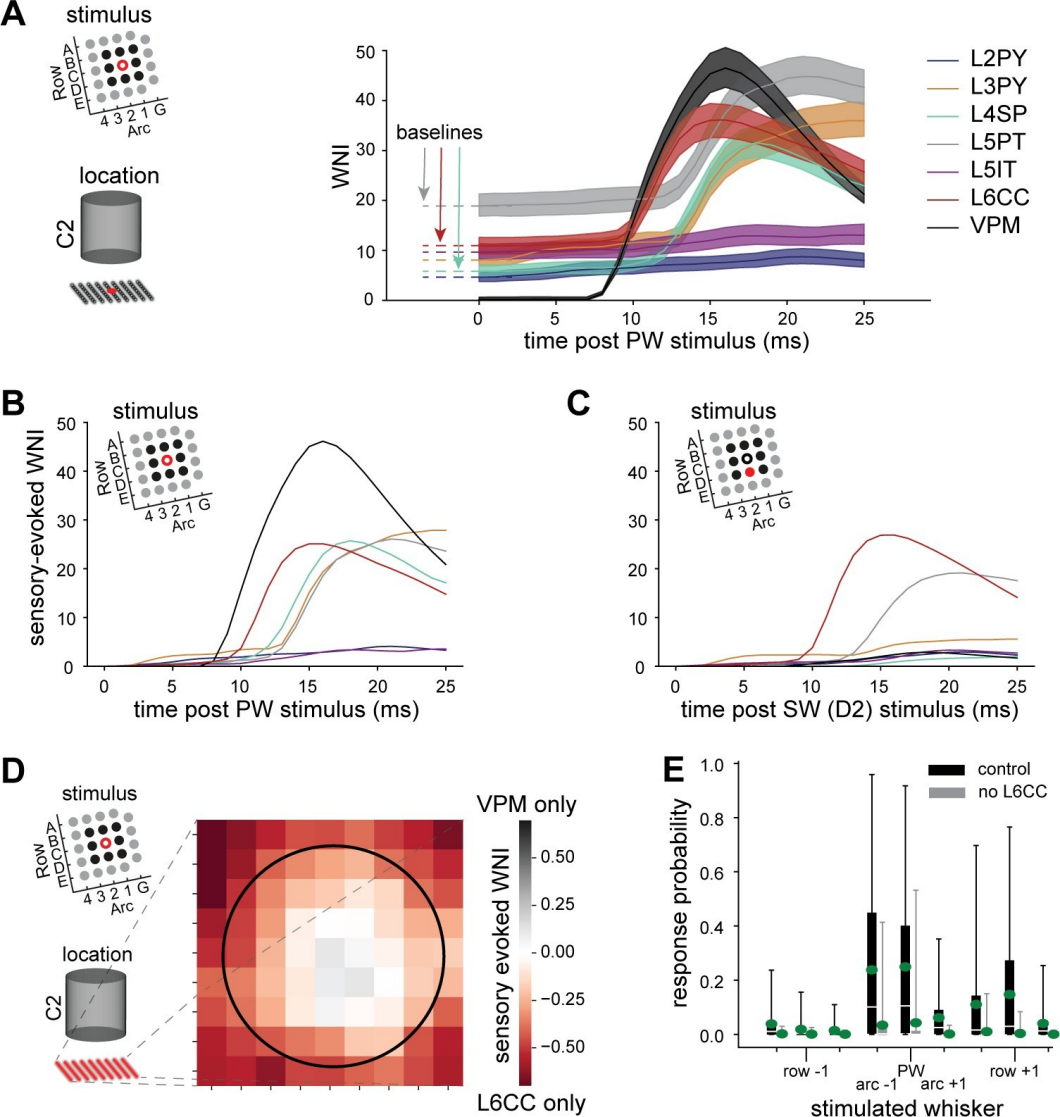
Reduced models predict contribution of input pathways to sensory responses. **A:** Absolute contribution of presynaptic populations to WNI following a PW stimulus to a model located at the center of the C2 column: pyramidal neurons in L2/3 (L2PY, L3PY), spiny neurons in L4 (L4SP), intratelencephalic neurons in L5 (L5IT), L5PT, corticocortical neurons at the L5/6 border (L6CC), and relay cells in the ventral posterior medial nucleus of thalamus (VPM). Despite the lack of sensory-evoked responses by L4PY, L6CT and L6INV [14], their contributions were considered in the overall WNI calculations (**Figs 2** and 3). **B:** Sensory-evoked contribution (i.e. absolute contribution minus baseline for each input pathway) of presynaptic populations to WNI following a PW stimulus. **C:** Sensory-evoked contribution of presynaptic populations to WNI following a SW stimulus. **D:** Contribution of the main input pathways–VPM and L6CC–depending on the soma location of the L5PT model in a 9x9 grid across the C2 column for a PW stimulus. The black circle denotes the C2 column border. **E:** Comparison between model responses to 9 different whisker stimuli (PW and 8 SW) under control conditions and when removing sensory-evoked input from L6CC. Removing evoked L6CC activity attenuates responses, in particular to surround whiskers.

Which presynaptic neuron populations underlie the variable AP responses? While so far, we only distinguished between excitatory and inhibitory cells, the network model provides information about the location and cell type of the neuron from which each active synapse originates [15], for both thalamocortical (i.e., VPM) and excitatory intracortical cell types: pyramidal neurons in L2/3/4 (L2PY, L3PY, L4PY), spiny neurons in L4 (L4SP), intratelencephalic neurons in L5 (L5IT), L5PT, corticocortical neurons at the L5/6 border (L6CC) and in deep L6 (L6INV), and corticothalamic neurons in L6 (L6CT). To dissect how these presynaptic populations contribute to AP output, we utilized the reduced models and calculated the WNI separately for each cell type across whisker stimuli and network embeddings (i.e., we applied the spatiotemporal filters to synaptic input from each presynaptic cell type one at a time, **Fig 5A**). First, we analyzed L5PTs embedded at the center of the barrel column that is somatotopically aligned with the PW defined in this study (i.e., C2). The analysis shows that presynaptic neurons contribute in two ways to APs: by their spontaneous activity preceding the stimulus (‘baseline WNI’) and/or by their increase in activity upon whisker deflection (’sensory-evoked WNI’). Predominantly, L5PTs contribute to the baseline WNI (see **Fig 5A** baseline), due to their high spontaneous firing rates [12,17]. To isolate the effect of the sensory-evoked WNI, we subtracted the baselines (**Fig 5B**). After the stimulus, VPM provides the first sensory-evoked contribution to the WNI, followed by L6CC, which is the first intracortical cell type that responds reliably to VPM input [14]. For a SW stimulus (**Fig 5C**), the situation is different: VPM contributes little to AP output, while L6CCs provide the main drive. Thus, the reduced models predict that depending on the stimulated whisker, thalamocortical and intracortical pathways–primarily VPM and L6CCs–contribute differently to the sensory-evoked response.

Could variability in input from these two pathways underlie variations in the receptive fields across L5PTs? We computed, which fraction of the WNI is provided by VPM versus L6CC depending on the network embedding. This analysis revealed that L5PTs receive different amounts of input from these two pathways depending on their location (**Fig 5D**). As a result, L6CCs provide strong input to L5PTs for both PW and SW stimuli, whereas VPM provides strong input for PW stimuli only. Inactivating L6CCs–a manipulation which we performed previously *in silico* and *in vivo* [14]–results in narrow receptive fields, as it largely abolishes L5PT responses to SWs, but it also reduces the probability of PW responses. We repeated this manipulation now with the reduced models. Indeed, inactivation of L6CCs resulted in a major reduction of all sensory responses, and a narrowing of receptive fields across all models and network embeddings (**Fig 5E**). Thus, the reduced models capture the origins of the fast and broadly tuned responses that L5PTs in barrel cortex elicit upon single whisker deflections–i.e., drive to proximal dendrites via the VPM➔L6CC➔L5PT pathway [14].

## Discussion

In this paper we introduce a computational approach for dissecting network-neuron interactions that underlie sensory-evoked responses in cortex. By deriving analytically tractable models from biologically-detailed multi-scale models of L5PTs in barrel cortex, we identify three features that are sufficient to predict the sensory responses and receptive fields that these neurons show *in vivo* upon passive deflections of single facial whiskers. By generating a GLM, which is based on these features, we demonstrate that the input-output computation is robust against variations in morphology and biophysical properties of the dendrites. Diversity in dendritic properties is hence predicted to have only a minor contribution to trial-to-trial and cell-to-cell variability of sensory-evoked responses and receptive fields. Instead, we show that variation in network input–i.e., from VPM thalamus and barrel cortex–is sufficient to account for the *in vivo* observed response variability.

The model reduction revealed network-neuron interactions underlying sensory-evoked responses of L5PTs. We recently showed that L6CCs, which respond first to sensory stimulation and which provide direct input to L5PTs, are required for the fast and broadly tuned responses of L5PTs [14]. This conclusion is also supported by the findings in this study. However, the drive by L6CCs does not imply that the upper layers cannot exert influence on the sensory-evoked responses of L5PTs. Instead, based on the present results we would predict that any inputs that neurons in the upper layers provide to the proximal dendrites of L5PTs, and which precede their response, will be integrated according to the input-output computation shown in this study. Thus, the upper layers can influence sensory-evoked responses of L5PTs, for example by providing inputs that are driven by non-sensory information streams, as has been suggested previously [18].

We want to emphasize that the reduced models are derived for one specific *in vivo* condition–passive deflections of single whiskers in anaesthetized rats. While we find that the input-output computation of L5PTs under this condition can be captured in very simple models, this is likely not the case in general. Previous studies have shown how to convert multi-compartmental models into deep artificial neural networks [19], simplified conductance based models [20–22] or stacks of linear-nonlinear units [23], which maintain high accuracy throughout a wide range of input conditions. All of these models are highly complex–for example Beniaguev et al. (2021) find that a 7-layer convolutional network is necessary to capture the immense computational power of a single neuron with NMDA synapses. Here, we have explored an orthogonal approach: by limiting the synaptic input conditions to those present in one specific experimental condition and thereby compromising on the generalizability of the model to other input scenarios, we can derive accurate yet analytically tractable models that reveal the simplest interpretation for how *in vivo* observed responses arise from the complex interplay between neuronal and network properties. Our focus on one specific *in vivo* condition was further motivated by a recent theoretical study which showed that synaptic input patterns can modify dendritic compartmentalization [24]. Thus, for the generation of interpretable models that accurately capture input-output computations across *in vivo* conditions, it is likely necessary to apply our reduction approach to each of these *in vivo* conditions separately.

Generating interpretable reduced models for another *in vivo* condition would require first a multi-scale model that accounts for the spatiotemporal input patterns and response variability of this condition. In this new condition, additional features and a more detailed description of passive and/or active properties of the dendrites may be required to accurately predict AP output. For example, based on passive properties, the shape of the EPSP evoked by a synapse activation depends on its distance to the soma, and neurons may exploit this to enhance their computational capabilities [25]. Additionally, both excitatory and inhibitory synapses can have a net inhibitory effect via shunting inhibition, which is not featured in our reduced models [2]. Furthermore, even though we demonstrate that the reduced models capture effects of NMDA in the properties of their spatial and temporal filters, more complex nonlinear model structures may be necessary to capture more complex *in vivo* input conditions. Similarly, dendrites have active properties by which they e.g. generate dendritic calcium APs that can modulate sensory responses with bursts of APs [26]. In awake animals, specifically the occurrence of such bursts is increased in the responses that L5PTs in barrel cortex elicit upon passive whisker deflections [27] and during active sensing [28,29]. The GLMs derived here cannot account for bursts (**S8 Fig**). Revisiting trials which are misclassified by the current reduced model and determining which additional features are needed to accurately predict burst responses would thereby result in a new minimal description of the input-output computation performed by neurons under this new experimental condition.

Many other modelling approaches exist that express input-output computations of single neurons. A popular choice for modelling how neurons respond to network input are leaky integrate-and-fire models, which are computationally efficient and highly interpretable but have no representation of the constituents of a biological neuron, and a priori *assume* the input-output computation. In contrast, our approach starts with a set of morphologically and biophysically diverse neuron models, and incorporates electrophysiological data to capture the characteristic dendritic and somatic properties. Next, the input-output computation is *derived* by exposing these detailed models to well-constrained spatiotemporal synaptic input patterns of one specific *in vivo* condition. The input-output computations are captured in reduced models which inherit the properties of the detailed models; in other words, the reduced models are equivalent to the detailed models thereby representing a biological neuron. Our reduced models are computationally as efficient as leaky integrate-and fire models, but are based on three features that are necessary and sufficient to capture the input-output computation of L5PTs for the investigated condition. As they are derived from morphologically and biophysically diverse models, they account for biological variability in dendritic properties. Thereby, the reduced models set the stage for simulations of networks with high biological realism at similar computational costs to integrate-and-fire networks.

While presently we rely on a multi-scale model as the basis for our reduction approach, in the coming years, large-scale voltage-imaging and connectome data at electron microscopic resolution may become available. Such experimental approaches could provide data at a similar level of detail to our multi-scale models. The reduction approach we present here can hence be readily applied to such future experimental data, and thereby facilitate interpretation of how the observed activity arises from the complex interplay between neuronal and network properties. Thus, our approach may provide a roadmap to reveal input-output computations and underlying network-neuron interactions across different *in vivo* conditions and for different cell types.

## Methods

### Ethics statement

No animal experiments were carried out in this study. The previously reported animal experiments [12], were carried out after evaluation and approval by the local German authorities, and in accordance with the animal welfare guidelines of the Max Planck Society.

### Extracellular recordings

We reported the data for receptive fields (RFs) and morphologies used in this study previously [12]. Briefly, Wistar rats (P25-30, both sexes) were anesthetized with urethane and responses to passive single whisker deflections (applied with a piezo manipulator) of the PW and SWs were recorded via a cell-attached pipette. The *in vivo* recorded neurons were filled with biocytin and morphologically reconstructed to identify their cell types. We determined the PW by identifying the barrel column in which the soma was located–i.e., the PW is not necessarily the one that evoked the strongest response. The *in vivo* data for L5PTs used in this study comprised exclusively L5PTs located in the D2 column.

### Morphological reconstructions

Neuronal structures were extracted from image stacks using a previously reported automated tracing software [30]. For reconstruction of biocytin labeled neurons, images were acquired using a confocal laser scanning system (Leica Application Suite Advanced Fluorescence SP5; Leica Microsystems). 3D image stacks of up to 2.5mm × 2.5mm × 0.05 mm were acquired at 0.092 × 0.092 × 0.5μm per voxel (63x magnification, NA 1.3). Image stacks were acquired for each of 45–48 consecutive 50μm thick tangential brain slices that range from the pial surface to the white matter. Manual proof-editing of individual sections, and automated alignment across sections were performed using custom-designed software [31]. Pia, barrel and white matter outlines were manually drawn on low-resolution images (4x magnification dry objective). Using these anatomical reference structures, all reconstructed morphologies were registered to a standardized 3D reference frame of rat vS1 [32].

### Multi-compartmental models

We selected 5 L5PT reconstructions that are representative of the morphological variability of this cell type. Multi-compartmental models were generated for these morphologies as described previously [14,16]. Briefly, a simplified axon morphology was attached to the soma of the reconstructed L5PT dendrite morphology [33]. The axon consisted of an axon hillock with a diameter tapering from 3μm to 1.75μm over a length of 20μm, an axon initial segment of 30μm length and diameter tapering from 1.75μm to 1μm diameter, and 1 mm of myelinated axon (diameter of 1μm). Next, a multi-objective evolutionary algorithm was used to find parameters for the passive leak conductance and the density of Hodgkin-Huxley type ion channels on soma, basal dendrite, apical dendrite and axon initial segment, such that the neuron model is able to reproduce characteristic electrophysiological responses to somatic and dendritic current injections of L5PTs within the experimentally observed variability, including back-propagating APs, calcium APs, and AP responses to prolonged somatic current injections [16]. We augmented the original biophysical model of L5PTs [14,16] with two ion channel parameters as previously described [34]: in accordance with a previous report [35], the density of the fast non-inactivating potassium channels (Kv3.1) was allowed to linearly decrease with soma distance until it reaches a minimum density (i.e., the slope and minimum density are two additional parameters, see [34]). The diameter of the apical dendrites was optimized by a scaling factor between 0.3 and 3. We incorporated the IBEA algorithm [36] for optimization. The optimization was terminated if there was no progress or when acceptable models had been found. We repeated the optimization process several times. From each independent run, we selected one model for which the maximal deviation from the experimental mean in units of standard deviation across all objectives was minimal (0.9–1.9 mean STDs across objectives).

### Network embeddings

Cell type-specific thalamocortical (from the ventral posteromedial thalamic nucleus (VPM) which is the primary thalamic nucleus of the whisker) and intracortical (from excitatory and inhibitory cells in vS1) connections are derived from an anatomically realistic circuit model of rat vS1 [13], a procedure, which has been described in detail previously [14]. We embedded the dendrite morphologies selected for multi-compartmental modelling in the network model at 81 locations within the cortical barrel column representing the C2 whisker, which is located approximately in the center of vS1. For all *in silico* data presented in this study, the PW is hence the C2 whisker. Thus, the morphologies of the *in vivo* recorded L5PTs were registered to the C2 column, while preserving their *in vivo* soma depths and laminar dendrite distributions that was observed empirically in the D2 column [32]. The locations were the column center, and equally spaced grid with a distance of 50μm between adjacent somata. For each of the 81 locations, we estimated the location of presynaptic neurons in VPM and vS1 that provide input to the respective L5PT (**Fig 1D**) and where along the L5PT dendrite they synapse (**Fig 1E**).

### Synapse models

Synapse models and synaptic parameters (rise and decay times, release probabilities, reversal potentials) were reported previously [14]. Briefly, conductance-based synapses were modeled with a double-exponential time course. Excitatory synapses contained both AMPA receptors (AMPARs) and NMDARs in 1:1 ratio. Inhibitory synapses contained GABA ARs. The peak conductance of excitatory synapses from different presynaptic cell types was determined by assigning the same peak conductance to all synapses of the same cell type, activating all connections of the same cell type (i.e., all synapses originating from the same presynaptic neurons) one at a time, and comparing parameters of the resulting unitary postsynaptic potential (uPSP) amplitude distribution (mean, median and maximum) for a fixed peak conductance with experimental measurements *in vitro* (IC input [37]) or *in vivo* (TC input [18]). The peak conductance for synaptic inputs from each cell type was systematically varied until the squared differences between parameters of the *in silico* and *in vitro/in vivo* uPSP amplitude distributions were minimized (i.e., the mean, median and maximum of the distributions were used, and mean and median were weighted twice relative to the maximum). This procedure was repeated for each multi-compartmental model using the connectivity model for the location in the center of the C2 column. The peak conductance at inhibitory synapses was fixed at 1nS [17].

### Synaptic input patterns

Synaptic input patterns to the L5PT model were estimated as described previously [14]. Briefly, we first chose the whisker stimulus. Then presynaptic neurons were grouped by their cell type and the column in which they are located. For each column, the relative position to the stimulated whisker was determined. Neurons in this column were activated based on the electrophysiologically recorded responses for this cell type and relative position. Each AP in a presynaptic neuron is registered at all synapses between the presynaptic neuron and the L5PT model without delay and may cause a conductance change, depending on the release probability of the synapse. Depending on the network embedding, neurons receive different ratios of excitatory and inhibitory inputs, which is not guaranteed to maintain functional E/I balance [38]. We therefore introduced a scaling factor for evoked inhibitory input strength, which we constrained based on the empirically observed PW response probabilities. The scaling factors ranged between 0.79 and 1.56 depending on the multi-compartmental model.

### Multi-compartmental model simulation data

Simulations were performed using Python 2.7, dask [39] and NEURON 7.4 [40]. We simulated 1000 trials for each of the 9 multi-compartmental models and each of the 81 locations and 9 whisker stimuli (PW, all 8 SWs). Additionally, the database contained 81000 trials of one 2^nd^ SW (E2), which was however not incorporated in the analysis. This results in 810000 simulation trials per multi-compartmental model, which reflects the effect of biophysics, morphology, network embedding and stimulus on sensory-evoked responses.

### Reduced model inference

We performed the reduced model inference for each multi-compartmental model separately. We split the multi-compartmental model dataset for the respective model into a training and test dataset (split ratio: 70% to 30%, respectively). Synapses are binned based on their time point of activation (1ms bins) and soma distance (50 micron bins). We excluded trials of the recent AP category (in which there was an AP in the last 50ms). Spatial and temporal filters were constructed as a weighted sum of basis functions *f*_*i*_ and *g*_*j*_. The soma distance dependent weight *w*_*z*_ and the time dependent weight *w*_*τ*_ of a synapse are given by

$$w_{\tau }=\sum_{i}a_{i}f_{i}(\tau )$$

$$w_{z}=\sum_{j}b_{j}g_{j}(z)$$

where *a*_*i*_ and *b*_*j*_ are free parameters.

We used raised cosine functions [41] as basis functions. The temporal basis functions *f*_*i*_ were:

$$f_{i}(\tau )=\frac{1}{2}\cos\left(k\cdot \log\left[\tau +c\right]-\phi_{i}\right)+\frac{1}{2}$$

for *τ* such that k·log(t + c) ϵ [*ϕ*_*i*_ – π, *ϕ*_*i*_ + π] and 0 elsewhere. Values used were k = 3, c = 5, and φ ϵ [3, 12]. Analogously, the spatial basis functions *g*_*i*_ were:

$$g_{j}(z)=\frac{1}{2}\cos\left(k\cdot \log\left[z+c\right]-\phi_{j}\right)+\frac{1}{2}$$

for z such that k·log(z + c) ϵ [*ϕ*_*i*_ – π, *ϕ*_*i*_ + π] and 0 elsewhere. Values used were k = 2, c = 1, and φ ϵ [1, 11]. The basis functions are visualized in **S9 Fig**.

Using the spatiotemporal filters, we compute a weighted sum of active synapses at a time point t, in the following referred to as weighted net input (WNI). The WNI is computed as follows:

$$WNI(t)=\sum_{\tau ,z}n_{z,t-\tau ,E}\cdot w_{\tau ,E}\cdot w_{z,E}+\sum_{\tau ,z}n_{z,t-\tau ,I}\cdot w_{\tau ,I}\cdot w_{z,I}$$

where, *τ* ϵ [0, 80ms] is the time before t, *z* ϵ [0, 1300μm] is the distance of the synapse from the soma, and *n*_*z*,*t–τ*_ is the number of active synapses at a given 1ms time and 50 micron soma distance bin. *w*_*τ*_ and *w*_*z*_ are the temporal and spatial filter, respectively. *n*, *w*_*τ*_ and *w*_*z*_ are split by synapse type, as indicated by the subscripts E (excitatory) and I (inhibitory).

To adjust the free parameters (*a*_*i*_ and *b*_*j*_ for excitatory and inhibitory input) which determine the shape of the spatial and temporal filters, we use a gradient-free optimization method (COBYLA, implemented in SciPy: [42]) to maximize the area under the receiver operating characteristic curve (AUROC, see section ‘Analysis’) between the WNI and AP output for a selected 1ms time bin. The 1ms time bin selected for the optimization is in the following referred to as the inference time point *t*_*inference*_. For each multi-compartmental model, we performed this optimization for *t*_*inference*_ ϵ [0,25]. We then manually selected one inference time point that resulted in reduced models which generalized well to other time points. The selected inference time point of each selected model is visualized in **S4 Fig**. Finally, we normalized the spatial and temporal filters such that the peak of the temporal filter is 1 for excitatory synapses and -1 for inhibitory synapses, and the value of the spatial filter for excitatory synapses at spatial bin 0 is 1.

In order to calculate the spiking probability for a given WNI value, we used these filters to calculate the WNI for all multi-compartmental model simulation trials from the training dataset at *ti*_*nference*_, and recorded whether a AP occurred at this time bin or not. The trials were then binned by WNI. The AP probability for each bin corresponds to the proportion of trials that produced an output AP in the multi-compartmental model simulation (**Fig 2C**). Bins with few data points were combined to ensure a minimum of ten data points per bin. Linear interpolation was used to find the spiking probability corresponding to any WNI value, and WNI values greater/smaller than values seen in the biophysical model simulation were assigned the highest/lowest spiking probability respectively. We hereafter refer to this as the “nonlinearity” function. In order to estimate the effect of recent APs on spiking probability, WNI values were plotted against the time since the previous AP (**Fig 2D**), and a boundary was drawn to best separate spiking from non-spiking trials as follows. We dropped points in the lowest 5% of all WNI values (to remove the effect of outliers) and then drew a boundary along the minimum remaining spiking WNI values. This boundary was normalized such that it is zero for time to previous AP → ∞ by subtracting the respective offset. We found this two-step procedure (first estimate spatiotemporal filters based on a dataset in which recent APs are filtered out, second determine the post AP penalty based on these filters) to be more robust than a joint inference of both. WNI with penalty applied maintains a high AUROC over all stimulus periods (**Fig 2F**), while the uncorrected AUROC drops after the sensory stimulus.

### Reduced model simulations

After the inference of the reduced models, we computed the WNI for all time points for which we wish to predict the occurrence of APs (in the manuscript referred to as a prediction time point). AP output was generated iteratively for one millisecond time bin at a time. For each time point, we computed the WNI by applying the temporal and spatial filter to the synaptic input of the preceding 80ms. The WNI was transformed to an AP probability based on the nonlinearity derived above. APs were randomly sampled based on this probability. If an AP occurred, the WNI of consecutive time points was updated based on the post AP penalty.

### Analysis

‘AP probability’ is used to denote the probability that an AP is evoked within a 1ms time bin. ‘Response probability’ denotes the probability that one or more APs are generated in the 25ms following a whisker stimulus. ‘Accuracy’ is the percentage of trials in which the multi-scale and reduced model agree on whether or not an AP is elicited in a 25ms window following the whisker stimulus. We used the SciPy function ‘metrics.roc_auc_score’ to compute the AUROC score, which by default uses the full range of values (i.e. thresholds are automatically chosen such that the false positive rate varies between 0% and 100%). Distributions of response probabilities were compared with the two-sided Kolmogorov-Smirnov test using the SciPy function ‘scipy.stats.ks_2samp’.

## Supporting information

S1 Fig Network embeddings at 81 positions in and around the C2 column of the five *in vivo* reconstructed morphologies. (TIF)

S2 Fig Cell type and stimulus specific activity used to constrain the multi-scale model. **A**: Average whisker receptive fields of intracortical and thalamic cell types. **B**: Average post-stimulus time histogram (PSTH) of intracortical cell types.
(TIF)

S3 Fig Selection of features and inference method. **A-D**: Spatiotemporal filters are robust, independent of the method used for estimating the filters and the selected input features. Our approach: Optimizing superposition of basis functions, LDA: linear discriminant analysis, SVM: linear support vector machine. **E**: Prediction accuracy for different input features and inference methods. Temporal (top row panel A): weights are assigned depending on the time point of activation of synapses (‘time before prediction time point’) independent of their soma distance. Temporal (prox & distal): Additionally, synapses are grouped in ‘proximal’ and ‘distal’ based on a soma distance cutoff of 500 micrometers. Temporal & cell type: Synapses are grouped by presynaptic cell type. (L2: Temporal & cell type (prox & dist): Additionally, synapses are grouped in ‘proximal’ and ‘distal’ based on a soma distance cutoff of 500 micrometers. Temporal & spatial: synapses are weighted depending on soma distance and time point. This configuration performed best and has been used throughout the main manuscript.
(TIF)

S4 Fig Spike timing in all multi-compartmental (black) versus corresponding reduced models (blue). Rows correspond to each multi-compartmental model. The first row corresponds to example 1 in Fig 1 and the reduced model in Fig 2. Columns are from left to right: PSTHs of multi-compartmental and reduced models for a PW stimulus, PSTHs of multi-compartmental and reduced models for a PW and the eight SW stimuli (vertical lines reflect inference time points–i.e., the time point on which the respective GLM was trained), raster plots of 20 example trials with a PW stimulus, deviation in ms between APs predicted by the reduced vs. multi-compartmental model for PW and SW stimuli.
(TIF)

S5 Fig Differences in the decay of the spatial filter can largely be explained by the diameter of the dendritic trunk. **A:** Excitatory spatial filters for the seven models, as in Fig 3. **B:** spatial filter height at the 15^th^ spatial bin (corresponding to a soma distance of 700 to 750 microns) versus the mean trunk diameter. **C:** as B, but for the diameter at the primary bifurcation point of the neuron.
(TIF)

S6 Fig Active nonlinear mechanisms in simulations of passive whisker deflection with biophysically detailed multi-compartmental models. **A:** example morphology 2 (from Fig 1), for which we re-simulated 1000 PW stimuli while recording synaptic AMPA and NMDA currents, ion currents and the transmembrane potential from the marked branches. **B:** example trial with a 2 AP burst response at the soma, a Ca-AP at the primary bifurcation point (BP), and AMPA and NMDA currents of an example synapse at the example branch. We quantified the charge exchanged through the AMPA and NMDA receptors of each synapse during the 25ms window following the whisker stimulus (‘response window’). **C:** synaptic currents of all synapses recorded on distal branches. Yellow lines are synapses on the example branch. **D:** as C, but for basal dendrites. **E:** Sodium currents recorded at distal dendritic branches. The yellow line is the example branch. **F:** Ratio of NMDA/AMPA area under the curve (AUC) across 1000 simulation trials.
(TIF)

S7 Fig NMDA influences sensory-evoked responses and input-output computation. **A**: Simulated receptive fields to passive single whisker deflections for one L5PT multi-compartmental model across 81 network embeddings, with NMDA conductance set to 100%, 50% and 0% of control value. **B**: Response probability to a principal whisker stimulus depending on the amount of NMDA. **C**: Spatial and temporal filters (red: excitatory synapses, blue: inhibitory synapses) inferred from multi-compartmental model with different amount of NMDA. **D-E**: Width of spatial and temporal filters depending on the amount of NMDA. **F**: AUROC score of reduced model (GLM) depending on the amount of NMDA in the multi-compartmental model.
(TIF)

S8 Fig AP counts are not well estimated by the reduced model. Predicted mean number of APs in response to a whisker stimulus for 7 multi-compartmental/reduced models at 81 different positions, for PW and 8 SW stimuli deviates from the multi-compartmental models. In comparison, response probability (See Fig 3) is very well captured by the reduced models. This indicates that the mechanisms discriminating single AP responses from burst responses are not well captured by the reduced model.
(TIF)

S9 Fig Basis functions for temporal (left) and spatial (right) filters. (TIF)

S1 Table Electrophysiological responses of the multi-compartmental models used in this study determined from the stimulus protocols shown in Fig 1H. (DOCX)

S2 Table Biophysical parameters of multi-compartmental models used in this study. (DOCX)

S3 Table Single trial accuracy of reduced models versus multi-compartmental models. **T**ables are confusion matrices representing the number of trials in which at least one AP was elicited in the response window (25ms post whisker stimulus) in the biophysically detailed multi-compartmental models (bio) and reduced models, respectively. Data is provided for all whisker stimuli, i.e., the principal and 8 surround whiskers. The shaded value is the overall accuracy (i.e., the percentage of simulation trials in which the reduced and biophysically detailed model match).
(DOCX)
