# Supplementary figures and images for: Network-neuron interactions underlying sensory responses of layer 5 pyramidal tract neurons in barrel cortex
Source: PLoS Comput Biol. 2024 Apr 16;20(4):e1011468. doi: 10.1371/journal.pcbi.1011468 (PMC11051592; doi:10.1371/journal.pcbi.1011468)

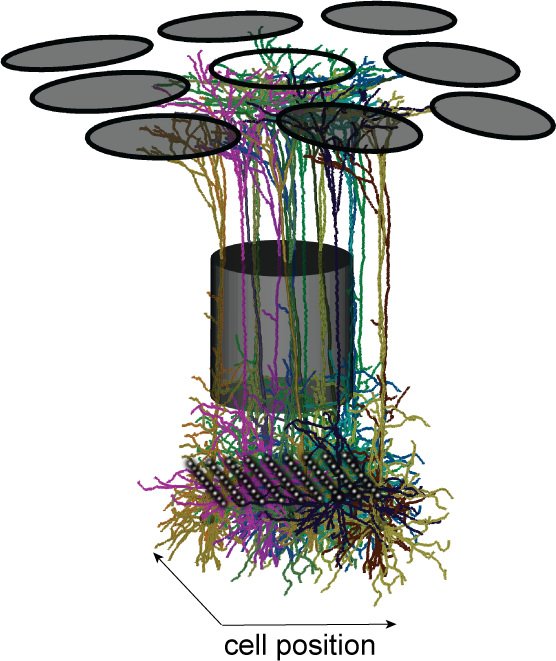

Supplement: S1 Fig — (TIF) [file pcbi.1011468.s001.tif]

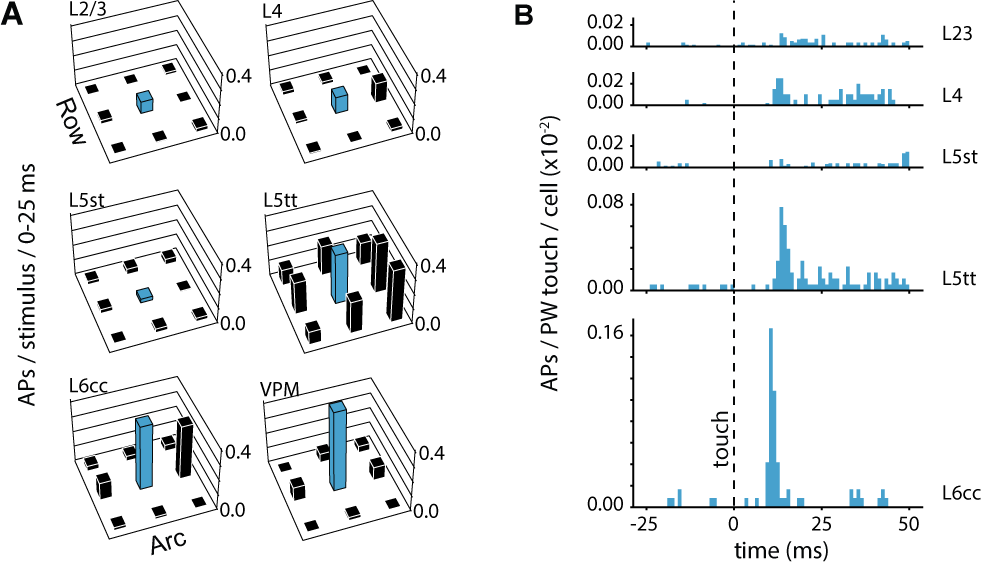

Supplement: S2 Fig — A: Average whisker receptive fields of intracortical and thalamic cell types. B: Average post-stimulus time histogram (PSTH) of intracortical cell types. (TIF) [file pcbi.1011468.s002.tif]

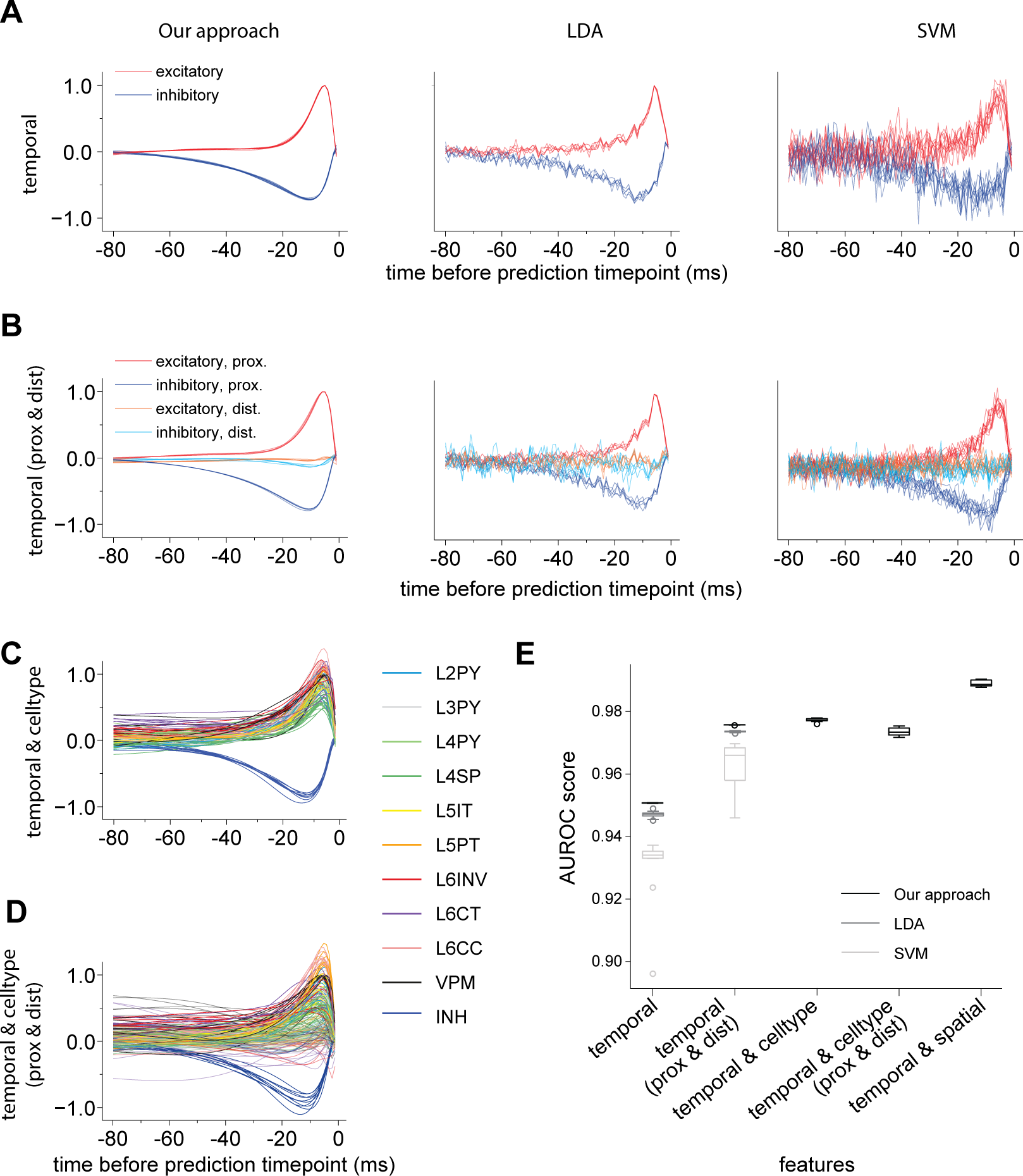

Supplement: S3 Fig — A-D: Spatiotemporal filters are robust, independent of the method used for estimating the filters and the selected input features. Our approach: Optimizing superposition of basis functions, LDA: linear discriminant analysis, SVM: linear support vector machine. E: Prediction accuracy for different input features and inference methods. Temporal (top row panel A): weights are assigned depending on the time point of activation of synapses (‘time before prediction time point’) independent of their soma distance. Temporal (prox & distal): Additionally, synapses are grouped in ‘proximal’ and ‘distal’ based on a soma distance cutoff of 500 micrometers. Temporal & cell type: Synapses are grouped by presynaptic cell type. (L2: Temporal & cell type (prox & dist): Additionally, synapses are grouped in ‘proximal’ and ‘distal’ based on a soma distance cutoff of 500 micrometers. Temporal & spatial: synapses are weighted depending on soma distance and time point. This configuration performed best and has been used throughout the main manuscript. (TIF) [file pcbi.1011468.s003.tif]

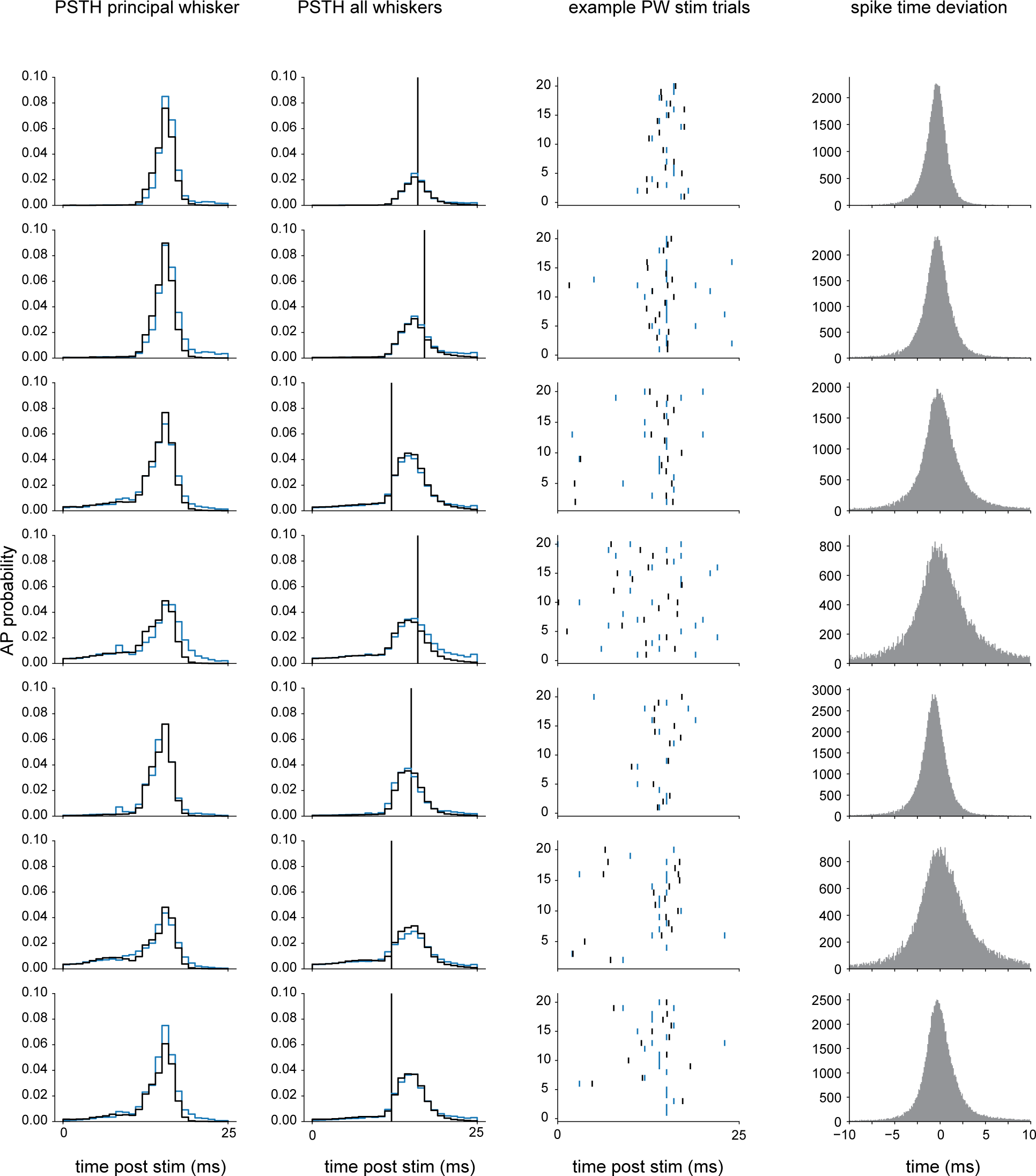

Supplement: S4 Fig — Rows correspond to each multi-compartmental model. The first row corresponds to example 1 in Fig 1 and the reduced model in Fig 2. Columns are from left to right: PSTHs of multi-compartmental and reduced models for a PW stimulus, PSTHs of multi-compartmental and reduced models for a PW and the eight SW stimuli (vertical lines reflect inference time points–i.e., the time point on which the respective GLM was trained), raster plots of 20 example trials with a PW stimulus, deviation in ms between APs predicted by the reduced vs. multi-compartmental model for PW and SW stimuli. (TIF) [file pcbi.1011468.s004.tif]

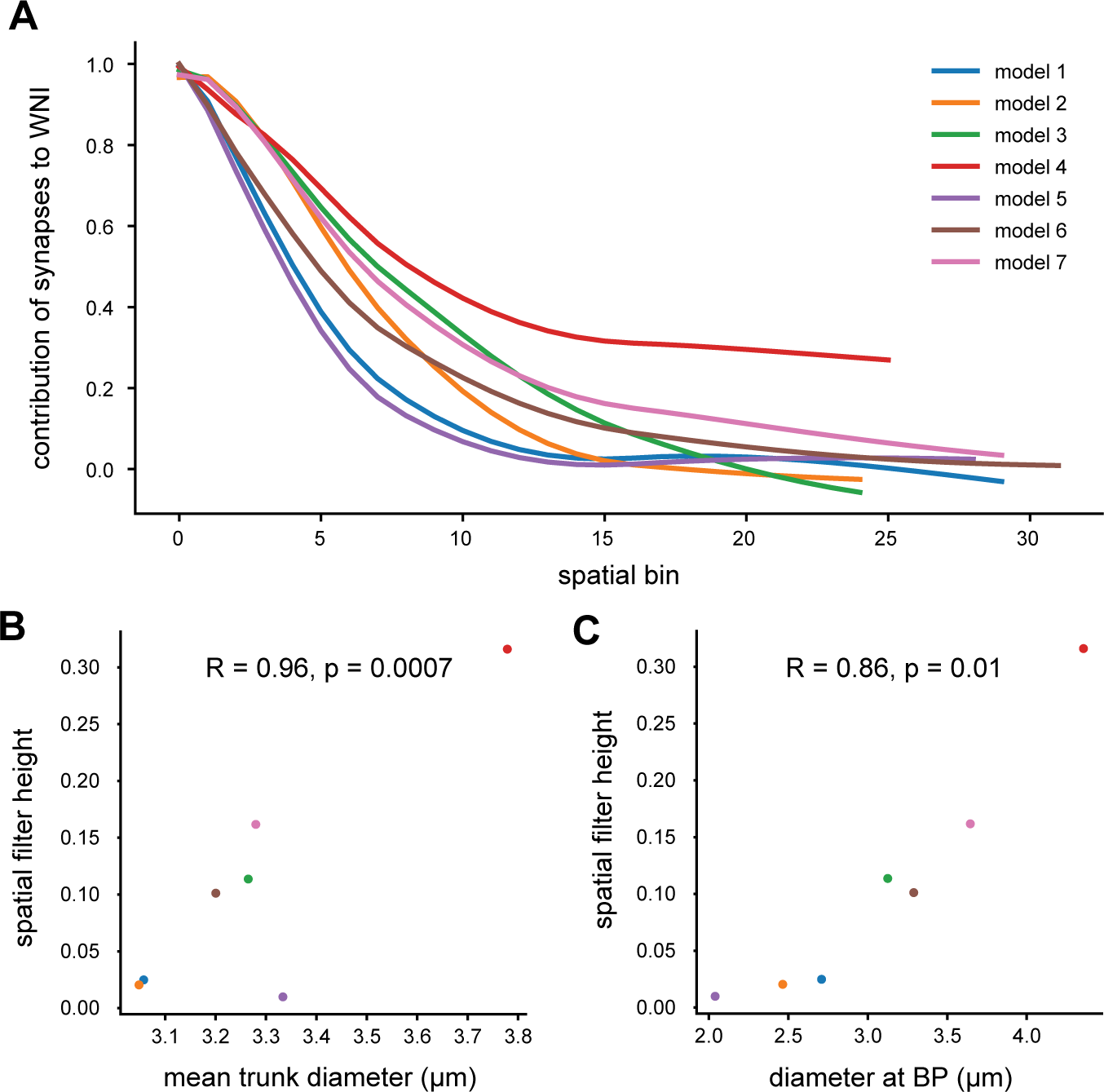

Supplement: S5 Fig — A: Excitatory spatial filters for the seven models, as in Fig 3. B: spatial filter height at the 15th spatial bin (corresponding to a soma distance of 700 to 750 microns) versus the mean trunk diameter. C: as B, but for the diameter at the primary bifurcation point of the neuron. (TIF) [file pcbi.1011468.s005.tif]

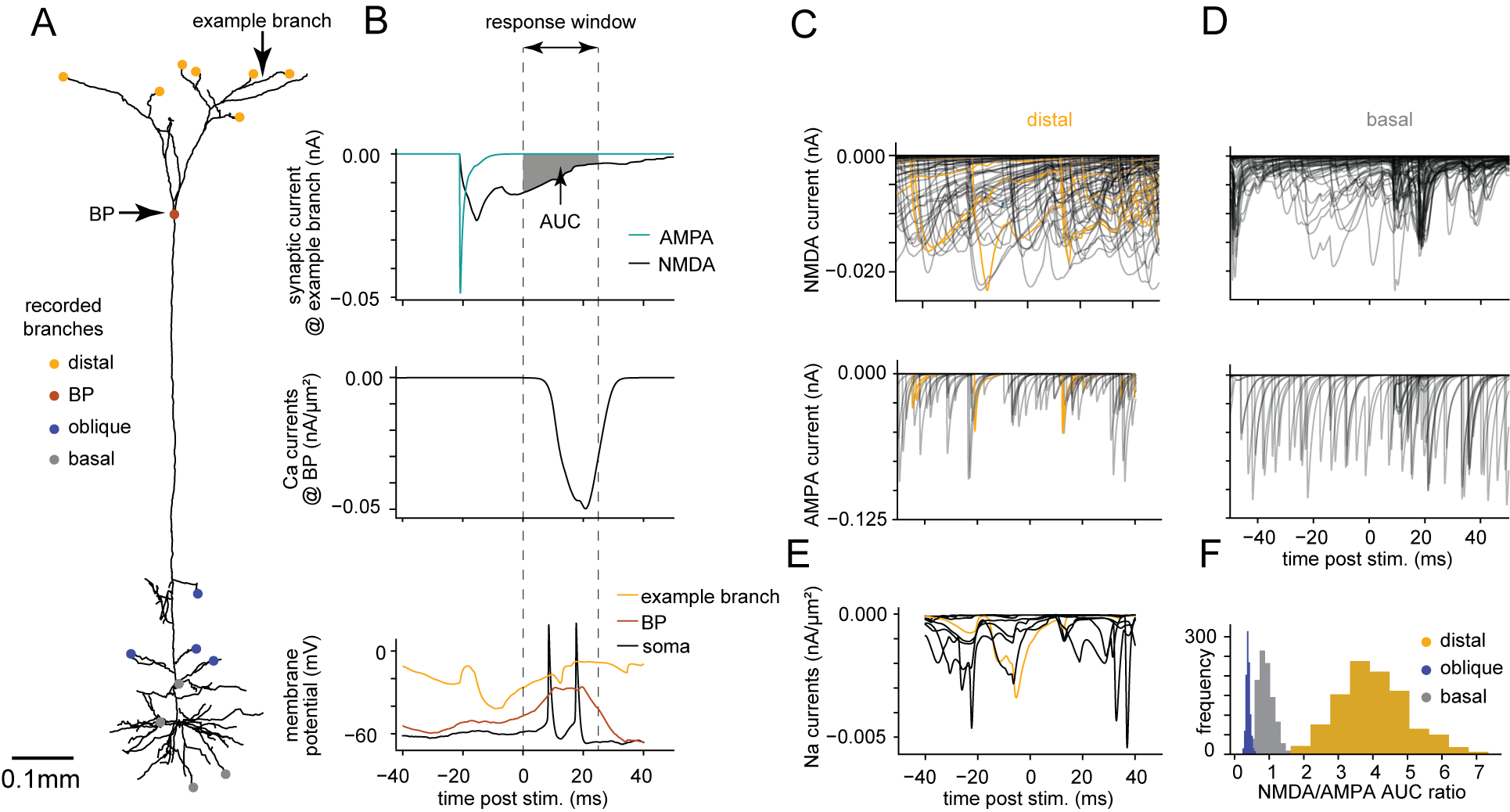

Supplement: S6 Fig — A: example morphology 2 (from Fig 1), for which we re-simulated 1000 PW stimuli while recording synaptic AMPA and NMDA currents, ion currents and the transmembrane potential from the marked branches. B: example trial with a 2 AP burst response at the soma, a Ca-AP at the primary bifurcation point (BP), and AMPA and NMDA currents of an example synapse at the example branch. We quantified the charge exchanged through the AMPA and NMDA receptors of each synapse during the 25ms window following the whisker stimulus (‘response window’). C: synaptic currents of all synapses recorded on distal branches. Yellow lines are synapses on the example branch. D: as C, but for basal dendrites. E: Sodium currents recorded at distal dendritic branches. The yellow line is the example branch. F: Ratio of NMDA/AMPA area under the curve (AUC) across 1000 simulation trials. (TIF) [file pcbi.1011468.s006.tif]

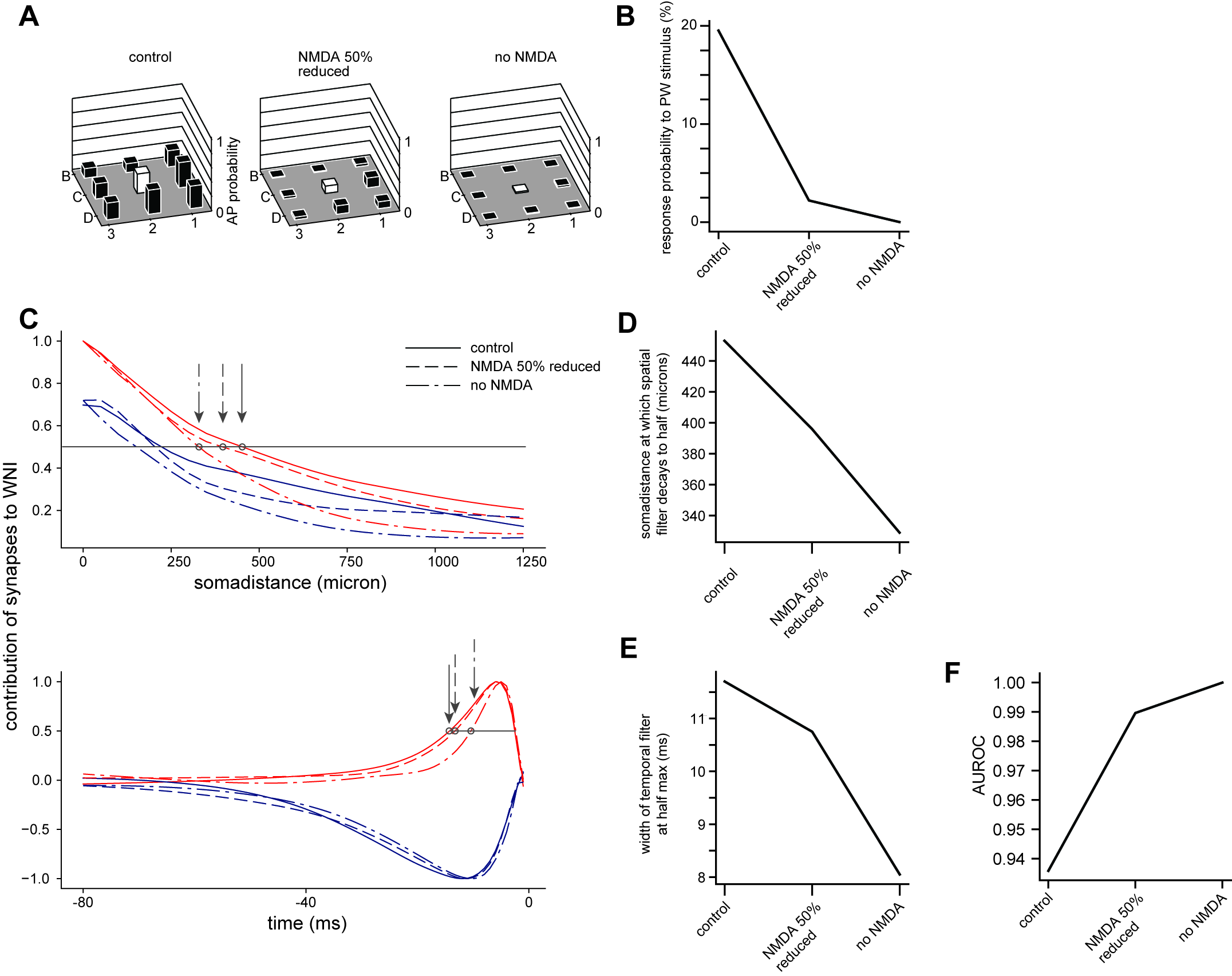

Supplement: S7 Fig — A: Simulated receptive fields to passive single whisker deflections for one L5PT multi-compartmental model across 81 network embeddings, with NMDA conductance set to 100%, 50% and 0% of control value. B: Response probability to a principal whisker stimulus depending on the amount of NMDA. C: Spatial and temporal filters (red: excitatory synapses, blue: inhibitory synapses) inferred from multi-compartmental model with different amount of NMDA. D-E: Width of spatial and temporal filters depending on the amount of NMDA. F: AUROC score of reduced model (GLM) depending on the amount of NMDA in the multi-compartmental model. (TIF) [file pcbi.1011468.s007.tif]

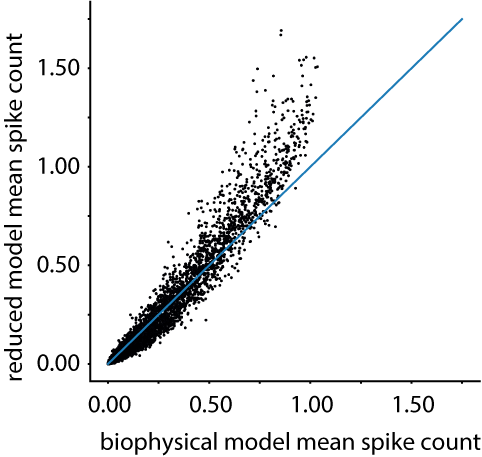

Supplement: S8 Fig — Predicted mean number of APs in response to a whisker stimulus for 7 multi-compartmental/reduced models at 81 different positions, for PW and 8 SW stimuli deviates from the multi-compartmental models. In comparison, response probability (See Fig 3) is very well captured by the reduced models. This indicates that the mechanisms discriminating single AP responses from burst responses are not well captured by the reduced model. (TIF) [file pcbi.1011468.s008.tif]

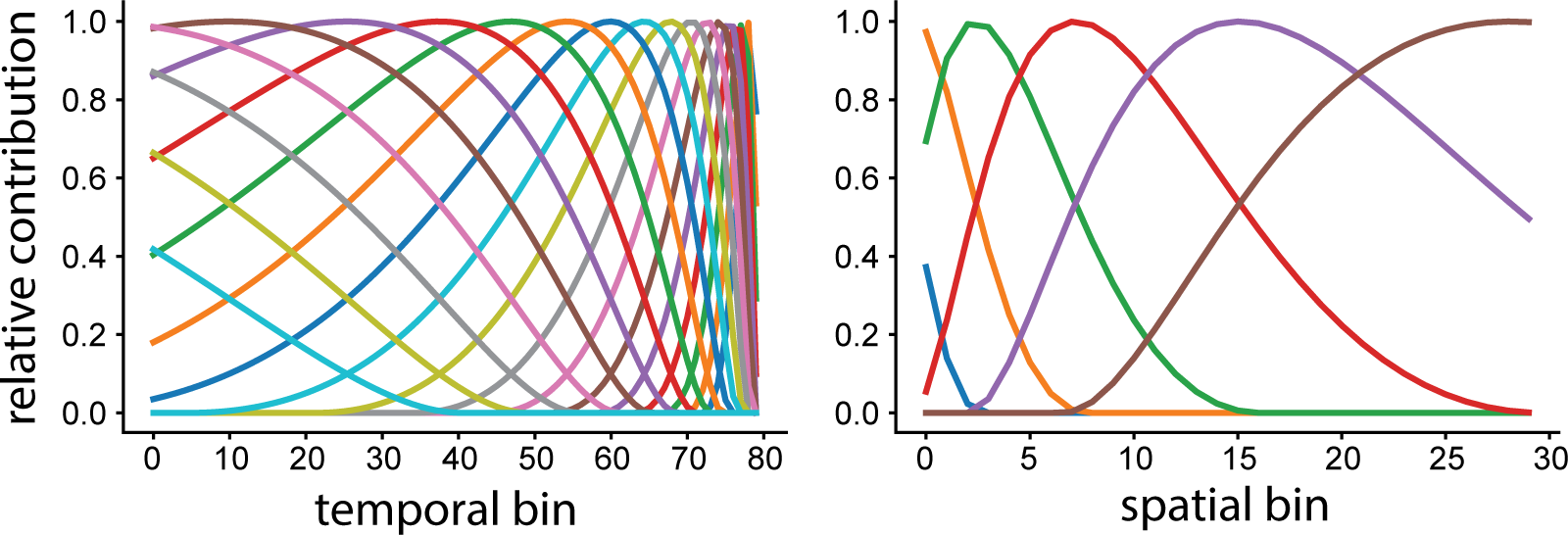

Supplement: S9 Fig — (TIF) [file pcbi.1011468.s009.tif]
