# Supplementary material for: Network-neuron interactions underlying sensory responses of layer 5 pyramidal tract neurons in barrel cortex
Source: PLoS Comput Biol. 2024 Apr 16;20(4):e1011468. doi: 10.1371/journal.pcbi.1011468 (PMC11051592; doi:10.1371/journal.pcbi.1011468)
Supplement: S1 Table — (DOCX) [file pcbi.1011468.s010.docx]

|  | **model 1** | **model 2** | **model 3** | **model 4** | **model 5** | **model 6** | **model 7** |
| --- | --- | --- | --- | --- | --- | --- | --- |
| **Somatic AP peak (mV)** | 36.79 | 31.89 | 33.81 | 30.28 | 36.70 | 39.49 | 32.65 |
| **Somatic AP half-width (ms)** | 1.08 | 0.96 | 1.02 | 1.10 | 1.16 | 1.03 | 1.00 |
| **bAP amplitue 180 microns below the BP** | 24.12 | 31.17 | 37.65 | 41.35 | 27.64 | 26.82 | 40.78 |
| **bAP amplitude at the BP** | 15.04 | 15.28 | 19.66 | 36.83 | 15.95 | 10.50 | 30.78 |
| **Somatic AP spike count** | 1 | 1 | 1 | 1 | 1 | 1 | 1 |
| **Mean AP height during 3 AP burst** | 28.91 | 22.24 | 27.25 | 25.59 | 26.44 | 31.99 | 24.60 |
| **Somatic AP ISI** | 10.29 | 10.85 | 10.71 | 11.46 | 10.30 | 12.03 | 11.87 |
| **Somatic AHP depth (mV)** | -62.20 | -62.79 | -63.54 | -64.54 | -62.45 | -65.45 | -62.65 |
| **Ca2+ AP peak (mV)** | 6.42 | 3.14 | 6.48 | 5.74 | -0.85 | 3.55 | 5.11 |
| **Ca2+ AP width (ms)** | 35.48 | 37.53 | 36.89 | 38.60 | 34.82 | 38.18 | 37.99 |
| **Somatic AP spike count (BAC stimulus)** | 3 | 3 | 3 | 3 | 3 | 3 | 3 |
| **First spike latency (ms), Step 1** | 29.96 | 44.92 | 32.81 | 27.14 | 28.23 | 33.90 | 31.53 |
| **First spike latency (ms), Step 2** | 18.23 | 21.98 | 19.94 | 17.85 | 17.19 | 20.24 | 19.69 |
| **First spike latency (ms), Step 3** | 6.61 | 7.03 | 7.27 | 7.15 | 5.68 | 6.75 | 7.32 |
| **Initial Burst ISI (ms), Step 1** | 11.91 | 67.17 | 42.92 | 23.50 | 15.05 | 48.00 | 15.59 |
| **Initial Burst ISI (ms), Step 2** | 8.75 | 16.49 | 19.06 | 14.49 | 9.26 | 22.17 | 10.62 |
| **Initial Burst ISI (ms), Step 3** | 5.78 | 6.03 | 6.44 | 8.43 | 5.61 | 6.06 | 6.04 |
| **AP peak (mV), Step 1** | 22.37 | 14.71 | 22.31 | 17.93 | 18.05 | 24.74 | 19.42 |
| **AP peak (mV), Step 2** | 22.23 | 14.30 | 22.20 | 18.05 | 17.64 | 24.80 | 18.56 |
| **AP peak (mV), Step 3** | 21.07 | 10.84 | 21.23 | 17.75 | 15.05 | 24.15 | 13.98 |
| **AP half-width (ms), Step 1** | 1.06 | 0.95 | 0.98 | 1.11 | 1.03 | 0.95 | 0.98 |
| **AP half-width (ms), Step 2** | 1.06 | 0.95 | 0.98 | 1.10 | 1.03 | 0.95 | 0.98 |
| **AP half-width (ms), Step 3** | 1.06 | 0.94 | 0.98 | 1.09 | 1.02 | 0.94 | 0.98 |
| **Fast AHP depth (mV), Step 1** | -60.10 | -59.82 | -61.33 | -61.89 | -59.58 | -62.05 | -60.55 |
| **Fast AHP depth (mV), Step 2** | -60.12 | -59.49 | -61.26 | -62.16 | -59.31 | -62.01 | -60.12 |
| **Fast AHP depth (mV), Step 3** | -59.64 | -56.78 | -60.66 | -62.36 | -57.77 | -61.62 | -57.46 |
| **Slow AHP depth (mV), Step 1** | -60.61 | -60.77 | -62.24 | -63.43 | -59.48 | -62.54 | -61.24 |
| **Slow AHP depth (mV), Step 2** | -60.49 | -60.28 | -61.98 | -63.43 | -59.04 | -62.51 | -60.68 |
| **Slow AHP depth (mV), Step 3** | -59.81 | -57.08 | -60.94 | -63.28 | -57.52 | -61.91 | -57.38 |
| **Slow AHP time, Step 1** | 0.33 | 0.16 | 0.20 | 0.30 | 0.29 | 0.20 | 0.32 |
| **Slow AHP time, Step 2** | 0.26 | 0.25 | 0.25 | 0.31 | 0.28 | 0.23 | 0.33 |
| **Slow AHP time, Step 3** | 0.22 | 0.27 | 0.22 | 0.22 | 0.20 | 0.26 | 0.15 |
| **AP frequency (Hz), Step 1** | 10.50 | 7.00 | 10.00 | 10.50 | 11.00 | 9.50 | 10.50 |
| **AP frequency (Hz), Step 2** | 15.00 | 13.50 | 14.00 | 13.50 | 15.50 | 13.50 | 14.00 |
| **AP frequency (Hz), Step 3** | 25.00 | 29.00 | 23.50 | 21.00 | 25.50 | 21.50 | 28.00 |
| **Adaptation index, Step 1** | 0.01 | 0.02 | 0.01 | -0.01 | 0.02 | 0.00 | 0.00 |
| **Adaptation index, Step 2** | 0.02 | 0.01 | 0.01 | 0.00 | 0.02 | 0.01 | 0.01 |
| **Adaptation index, Step 3** | 0.01 | 0.01 | 0.01 | 0.01 | 0.01 | 0.01 | 0.01 |
| **ISI-CV, Step 1** | 0.12 | 0.09 | 0.08 | 0.07 | 0.12 | 0.05 | 0.06 |
| **ISI-CV, Step 2** | 0.16 | 0.12 | 0.09 | 0.06 | 0.13 | 0.09 | 0.12 |
| **ISI-CV, Step 3** | 0.15 | 0.13 | 0.10 | 0.12 | 0.14 | 0.13 | 0.13 |
