# Supplementary material for: Network-neuron interactions underlying sensory responses of layer 5 pyramidal tract neurons in barrel cortex
Source: PLoS Comput Biol. 2024 Apr 16;20(4):e1011468. doi: 10.1371/journal.pcbi.1011468 (PMC11051592; doi:10.1371/journal.pcbi.1011468)
Supplement: S2 Table — (DOCX) [file pcbi.1011468.s011.docx]

| Parameter name/Model ID | **1** | **2** | **3** | **4** | **5** | **6** | **7** |
| --- | --- | --- | --- | --- | --- | --- | --- |
| **CaDynamics_E2.apic.decay** | 121.0674 | 138.7704 | 188.4791 | 114.9947 | 124.7849 | 94.58709 | 124.0039 |
| **CaDynamics_E2.apic.gamma** | 0.000813 | 0.000551 | 0.000522 | 0.000579 | 0.000519 | 0.000567 | 0.003474 |
| **CaDynamics_E2.axon.decay** | 119.401 | 333.7151 | 346.1883 | 196.5606 | 912.4342 | 973.4132 | 222.7396 |
| **CaDynamics_E2.axon.gamma** | 0.001766 | 0.002154 | 0.001582 | 0.000642 | 0.004945 | 0.000515 | 0.038612 |
| **CaDynamics_E2.soma.decay** | 301.457 | 530.8009 | 327.2006 | 155.3969 | 311.9493 | 213.7342 | 88.21946 |
| **CaDynamics_E2.soma.gamma** | 0.000517 | 0.000554 | 0.010279 | 0.007425 | 0.000502 | 0.03446 | 0.001622 |
| **Ca_HVA.apic.gCa_HVAbar** | 0.002759 | 0.004131 | 0.003894 | 0.001015 | 0.004717 | 0.003176 | 0.001476 |
| **Ca_HVA.axon.gCa_HVAbar** | 0.000185 | 0.000488 | 2.86E-05 | 0.000819 | 2.96E-05 | 2.85E-05 | 5.13E-05 |
| **Ca_HVA.soma.gCa_HVAbar** | 0.000683 | 0.000298 | 3.56E-05 | 0.00011 | 0.000564 | 2.22E-05 | 0.000502 |
| **Ca_LVAst.apic.gCa_LVAstbar** | 0.001881 | 0.063474 | 0.025106 | 0.002194 | 0.009637 | 0.001959 | 0.024519 |
| **Ca_LVAst.axon.gCa_LVAstbar** | 0.009016 | 0.006404 | 0.004188 | 0.003719 | 3.58E-05 | 0.00286 | 0.00011 |
| **Ca_LVAst.soma.gCa_LVAstbar** | 0.004735 | 0.002137 | 0.001545 | 0.000614 | 0.006072 | 5.14E-06 | 0.008861 |
| **Im.apic.gImbar** | 0.000113 | 4.52E-05 | 2.47E-05 | 2.7E-06 | 0.000381 | 4.03E-05 | 9.71E-07 |
| **K_Pst.axon.gK_Pstbar** | 0.004625 | 0.296952 | 0.290188 | 0.005763 | 0.104922 | 0.172764 | 0.077452 |
| **K_Pst.soma.gK_Pstbar** | 0.005713 | 0.078117 | 0.140957 | 0.003549 | 0.025351 | 0.152812 | 0.009897 |
| **K_Tst.axon.gK_Tstbar** | 0.035837 | 0.006818 | 0.096493 | 0.044702 | 0.03351 | 0.027037 | 0.040092 |
| **K_Tst.soma.gK_Tstbar** | 0.033603 | 0.003949 | 0.078375 | 0.099119 | 0.081466 | 0.075429 | 0.078967 |
| **NaTa_t.apic.gNaTa_tbar** | 0.016966 | 0.01891 | 0.017893 | 0.013856 | 0.016047 | 0.020064 | 0.017836 |
| **NaTa_t.axon.gNaTa_tbar** | 3.863664 | 3.553574 | 3.560277 | 3.899617 | 3.711704 | 0.109311 | 3.348559 |
| **NaTa_t.soma.gNaTa_tbar** | 3.208129 | 1.833618 | 3.362703 | 3.896084 | 1.325818 | 3.843608 | 3.992573 |
| **Nap_Et2.axon.gNap_Et2bar** | 0.009615 | 0.002898 | 0.001821 | 0.009965 | 0.002535 | 0.002898 | 0.009859 |
| **Nap_Et2.soma.gNap_Et2bar** | 0.002371 | 0.003883 | 0.009257 | 0.005123 | 0.000599 | 0.000328 | 0.001849 |
| **SK_E2.apic.gSK_E2bar** | 0.002111 | 0.002975 | 0.003351 | 0.001143 | 0.003455 | 0.003749 | 0.000585 |
| **SK_E2.axon.gSK_E2bar** | 0.009162 | 0.01546 | 0.077013 | 0.014094 | 0.08137 | 0.00117 | 0.020578 |
| **SK_E2.soma.gSK_E2bar** | 0.079714 | 0.029528 | 0.024539 | 0.066667 | 0.080914 | 0.059734 | 0.003856 |
| **SKv3_1.apic.gSKv3_1bar** | 0.002863 | 0.014618 | 0.01143 | 7.1E-05 | 0.007639 | 0.003849 | 8.58E-06 |
| **SKv3_1.apic.offset** | 0.703146 | 0.679136 | 0.594989 | 0.086458 |  | 0.845104 | 0.212368 |
| **SKv3_1.apic.slope** | -2.73404 | -2.04156 | -2.79123 | -2.98351 |  | -0.87599 | -1.09782 |
| **SKv3_1.axon.gSKv3_1bar** | 0.002863 | 0.014618 | 0.01143 | 7.1E-05 | 0.007639 | 0.003849 | 8.58E-06 |
| **SKv3_1.soma.gSKv3_1bar** | 0.002863 | 0.014618 | 0.01143 | 7.1E-05 | 0.007639 | 0.003849 | 8.58E-06 |
| **apic.g_pas** | 7E-05 | 5.77E-05 | 3.43E-05 | 4.54E-05 | 4.16E-05 | 3.37E-05 | 3.54E-05 |
| **axon.g_pas** | 2.42E-05 | 3.57E-05 | 2.09E-05 | 2.07E-05 | 4.99E-05 | 2.69E-05 | 3.49E-05 |
| **dend.g_pas** | 5.3E-05 | 8.21E-05 | 7.97E-05 | 4.15E-05 | 6.45E-05 | 6.26E-05 | 6.19E-05 |
| **soma.g_pas** | 2.31E-05 | 2.04E-05 | 4.96E-05 | 2.25E-05 | 2.54E-05 | 3.56E-05 | 2.01E-05 |
| **scale_apical** | 1 | 2.181484 | 2.766078 | 1.713925 | 1 | 2.910863 | 1.345393 |
