# Supplementary material for: Network-neuron interactions underlying sensory responses of layer 5 pyramidal tract neurons in barrel cortex
Source: PLoS Comput Biol. 2024 Apr 16;20(4):e1011468. doi: 10.1371/journal.pcbi.1011468 (PMC11051592; doi:10.1371/journal.pcbi.1011468)
Supplement: S3 Table — Tables are confusion matrices representing the number of trials in which at least one AP was elicited in the response window (25ms post whisker stimulus) in the biophysically detailed multi-compartmental models (bio) and reduced models, respectively. Data is provided for all whisker stimuli, i.e., the principal and 8 surround whiskers. The shaded value is the overall accuracy (i.e., the percentage of simulation trials in which the reduced and biophysically detailed model match). (DOCX) [file pcbi.1011468.s012.docx]

| model 1 |  | **AP (bio)** | **no AP (bio)** | **%** | model 5 |  | **AP (bio)** | **no AP (bio)** | **%** |
| --- | --- | --- | --- | --- | --- | --- | --- | --- | --- |
|  | **AP (reduced)** | 61348 | 13092 | 82.41 |  | **AP (reduced)** | 108704 | 23461 | 82.25 |
|  | **no AP (reduced)** | 10660 | 643900 | 98.37 |  | **no AP (reduced)** | 23444 | 573391 | 96.07 |
|  | **%** | 85.20 | 98.01 | 96.74 |  | **%** | 82.26 | 96.07 | 93.57 |
|  |  |  |  |  |  |  |  |  |  |
| model 2 |  | **AP (bio)** | **no AP (bio)** | **%** | model 6 |  | **AP (bio)** | **no AP (bio)** | **%** |
|  | **AP (reduced)** | 91659 | 23253 | 79.76 |  | **AP (reduced)** | 124309 | 38886 | 76.17 |
|  | **no AP (reduced)** | 21801 | 592287 | 96.45 |  | **no AP (reduced)** | 57407 | 508398 | 89.85 |
|  | **%** | 80.79 | 96.22 | 93.82 |  | **%** | 68.41 | 92.89 | 86.79 |
|  |  |  |  |  |  |  |  |  |  |
| model 3 |  | **AP (bio)** | **no AP (bio)** | **%** | model 7 |  | **AP (bio)** | **no AP (bio)** | **%** |
|  | **AP (reduced)** | 163240 | 30828 | 84.11 |  | **AP (reduced)** | 127239 | 23081 | 84.65 |
|  | **no AP (reduced)** | 52914 | 482018 | 90.11 |  | **no AP (reduced)** | 37475 | 541205 | 93.52 |
|  | **%** | 75.52 | 93.99 | 88.51 |  | **%** | 77.25 | 95.91 | 91.69 |
|  |  |  |  |  |  |  |  |  |  |
| model 4 |  | **AP (bio)** | **no AP (bio)** | **%** |  |  |  |  |  |
|  | **AP (reduced)** | 127538 | 57207 | 69.03 |  |  |  |  |  |
|  | **no AP (reduced)** | 52427 | 491828 | 90.37 |  |  |  |  |  |
|  | **%** | 70.87 | 89.58 | 84.96 |  |  |  |  |  |
